# Split-APEX implicates splicing factor SRSF1 and splicing helicases in ribosomal biogenesis

**DOI:** 10.3389/fmolb.2025.1714378

**Published:** 2025-12-19

**Authors:** Vasileios Paschalis, Max F. K. Wills, Philippe De Gusmao Araujo, Christian Lucas, Sumera Tubasum, Shijie Cui, Hesna Kara, Carlos Bueno-Alejo, Marina Santana-Vega, Andrea Taladriz-Sender, Zhengyun Zhao, Alexander Axer, Cyril Dominguez, Alasdair W. Clark, Glenn A. Burley, Andrew J. Hudson, Ian C. Eperon

**Affiliations:** 1 Institute for Structural and Chemical Biology, University of Leicester, Leicester, United Kingdom; 2 Department of Molecular and Cell Biology, University of Leicester, Leicester, United Kingdom; 3 James Watt School of Engineering, Advanced Research Centre, University of Glasgow, Glasgow, United Kingdom; 4 Department of Pure & Applied Chemistry and the Strathclyde Centre for Molecular Bioscience, University of Strathclyde, Glasgow, United Kingdom; 5 School of Chemistry, University of Leicester, Leicester, United Kingdom

**Keywords:** RNA splicing helicases, split-APEX, SRSF1, ribosomal biogenesis, spliceosomal assembly

## Abstract

SR proteins are RNA-binding proteins with one or two RNA recognition motif (RRM)-type RNA-binding domains and a C-terminal region rich in arginine-serine dipeptides. They function in cellular processes ranging from transcription to translation. The best-known SR protein, SRSF1, modulates RNA splicing by stabilizing the binding of constitutive splicing factors, but there is also evidence that it participates in constitutive splicing reactions and is present in spliceosomal complexes. It has been shown recently that it interacts with DDX23, an RNA helicase that triggers the transition from complex pre-B to complex B during activation of the spliceosome. To identify in which other steps of spliceosome assembly and reaction it might be present, we have used split-APEX with SRSF1 and a number of helicases, each of the latter being involved in a particular step. Peroxidase activity should only be reconstituted if SRSF1 and the helicase were in contact, and the consequent biotinylation should reveal proteins in the vicinity. Our results show that all the helicases tested can complement SRSF1, but that the proximal proteins are very similar in all cases. Moreover, the proteins identified fall into two major classes: splicing-related proteins and ribosomal proteins. The results raise the possibility that SRSF1 and the canonical helicases have hitherto unsuspected collaborative roles in ribosomal assembly or translation.

## Introduction

SRSF1 was first identified as both an essential splicing factor, required to complement cytoplasmic extracts to enable them to splice pre-mRNA *in vitro* (Krainer et al., 1990), and an alternative splicing factor involved in 5′SS selection (Ge and Manley, 1990), but it has been shown subsequently to have roles also in transcription, nuclear export, control of translation, nonsense-mediated decay, X-chromosome inactivation and genome stability (Arif et al., 2023; Das and Krainer, 2014; Ji et al., 2013; Long and Caceres, 2009; Maslon et al., 2014; Muller-McNicoll et al., 2016; Paz et al., 2014; Paz et al., 2021; Sliskovic et al., 2022; Trotman et al., 2025). Interactome studies have shown that SRSF1 is promiscuous: it associates directly or indirectly with over 30 other spliceosomal proteins(Akerman et al., 2015), as well as a number of proteins involved in other processes (Das and Krainer, 2014), and BioGRID lists 590 interactions.

The best-understood action of SRSF1 is as an activator of exon inclusion during splicing. It binds purine-rich enhancer sequences (Anczukow et al., 2015; Clery et al., 2021; Lavigueur et al., 1993; Sanford et al., 2009; Sun et al., 1993; Tacke and Manley, 1995) and stabilizes the binding of core splicing components at the 5′and 3′splice sites via protein-protein interactions (Eperon et al., 1993; Eperon et al., 2000; Kohtz et al., 1994; Lavigueur et al., 1993; Staknis and Reed, 1994; Tarn and Steitz, 1995; Wang and Manley, 1995; Wu and Maniatis, 1993). We have inferred from single molecule experiments that there is a low probability that an ESE is occupied at any moment by SRSF1, but that interactions with the splicing components by 3D diffusion stabilize a complex (Jobbins et al., 2018). Interestingly, however, SRSF1 can also be recruited to 5′splice sites by U1 snRNPs (Jamison et al., 1995; Jobbins et al., 2022), to which it binds by protein-RNA and protein-protein interactions (Jobbins et al., 2022; Kohtz et al., 1994; Paul et al., 2024; Xiao and Manley, 1998). This interaction may facilitate exon definition by a 5′SS, if the bound SRS1 interacts across the exon, or it may stabilize U1 snRNP binding to the pre-mRNA and thereby affect 5′splice site selection (Eperon et al., 1993; Jobbins et al., 2022).

Other findings suggest that SRSF1 may have a role in the spliceosome itself. SRSF1 RRM2 has been found in structures of the pre-B^act^ spliceosome (Townsend et al., 2020). Recent work has shown that there is also a direct interaction between SRSF1 and DDX23 (Segovia et al., 2024), a DEAD-box helicase that enters the spliceosome with the tri-snRNP and displaces the U1 snRNP during the pre-B to B complex transition (Charenton et al., 2019; Zhang Z. et al., 2024). In this case, the interaction of SRSF1 with both the U1 snRNP and DDX23 might facilitate docking of the U1 snRNP and the 5′splice site to DDX23 in the tri-snRNP in the pre-B complex, prior to displacement of the U1 snRNP. The role of SRSF1 may extend beyond the early stages alone, though, since the addition of SRSF1 or an RNA-tethered RS domain to a cytoplasmic extract led to contacts between the RS domain and the 5′splice site, branchpoint or 5′exon of the pre-mRNA in spliceosomal complexes A, B and C (Shen and Green, 2004; 2007). If SRSF1 does contact DDX23 in the spliceosome and remains after DDX23 leaves, we reasoned that it might come into very close proximity with the succession of other helicases that drive spliceosomal transitions and reactions (Dorner and Hondele, 2024; Enders et al., 2023; Wan et al., 2019; Wilkinson et al., 2020). Some support for this possibility comes from high-throughput screens that have linked SRSF1 with a number of helicases (Havugimana et al., 2012; Hein et al., 2015; Huttlin et al., 2021).

Molecular proximity can be tested by split-APEX, in which ascorbate peroxidase activity is reconstituted from two fragments fused to other proteins when the fragments are brought together by intermolecular interactions (Han et al., 2019). If biotin tyramide is supplied, peroxidase activity would generate short-lived radicals that biotinylate nearby proteins. Thus, the detection of biotinylated proteins would confirm that the two fragments of APEX2 had been brought together to enable the full protein to be reconstituted and, in addition, would provide an indication of the local protein environment when reconstitution occurs. We describe results here that show that SRSF1 does interact with helicases involved at all stages of pre-mRNA splicing, but these interactions take place in very similar environments, not necessarily in the spliceosome, and raise the possibility that SRSF1 and the helicases are involved in ribosomal biogenesis.

## Results

Split-APEX involves the separate fusion of two portions of a modified soybean ascorbate peroxidase (APEX2) to the two proteins being interrogated. The N-terminal and C-terminal portions (AP and EX respectively) are ∼200 and 50 amino acids in length, and had been selected for a low affinity for each other (Huttlin et al., 2021). They were cloned at the N-terminal side of full-length SRSF1, DDX46, DDX23, DHX16, DHX38, DHX8 and, as controls for intranuclear location, the speckle factor SRRM2 (Ilik et al., 2020) and the nuclear export factor TNPO3, the latter of which associates directly with SRSF1 during nuclear import (Maertens et al., 2014) (Supplementary Figure S1). DDX46 (an othologue of yeast Prp5) is a component of the U2 snRNP, associated with SF3B1 and plays critical roles in complex A formation (Perriman et al., 2003; Talkish et al., 2019; Yang et al., 2023; Zhang X. et al., 2024; Zhang et al., 2020); DDX23 (yeast Prp28) is a component of the tri-snRNP, required for displacement of the U1 snRNP during the transition from the pre-B to the B complex (Boesler et al., 2016; Charenton et al., 2019; Staley and Guthrie, 1999; Zhang Z. et al., 2024); DHX16 (yeast Prp2), in conjunction with the helicase Aquarius, is required for the step in assembly during which the branch site enters the active site and the catalytic B*/C complex forms (Kim and Lin, 1996; King and Beggs, 1990; Krishnan et al., 2013; Schmitzova et al., 2023; Zhan et al., 2024); DHX38 (yeast Prp16) replaces DHX16 in the spliceosome and rearranges the active site from a step 1 configuration in complex C to that required for step 2 in complex C* (Bertram et al., 2017; Fica et al., 2017; Schwer and Guthrie, 1992; Semlow et al., 2016; Wilkinson et al., 2021; Zhan et al., 2024; Zhan et al., 2018); DHX8 (yeast Prp22) replaces DHX38, and triggers dissociation of the spliced mRNA, leaving a spliceosome containing the lariat intron (Bertram et al., 2017; Company et al., 1991; Fica et al., 2017; Liu et al., 2017; McPheeters and Muhlenkamp, 2003; Schwer, 2008; Zhan et al., 2022); DDX48 (eIF4A3) plays an important role in assembling the post-splicing exon junction complex (Chan et al., 2004; Gehring et al., 2005). All the proteins were fused separately to both the AP and the EX fragments of APEX2. An additional control included the fusion of intact APEX2 to SRSF1.

**FIGURE 1:**
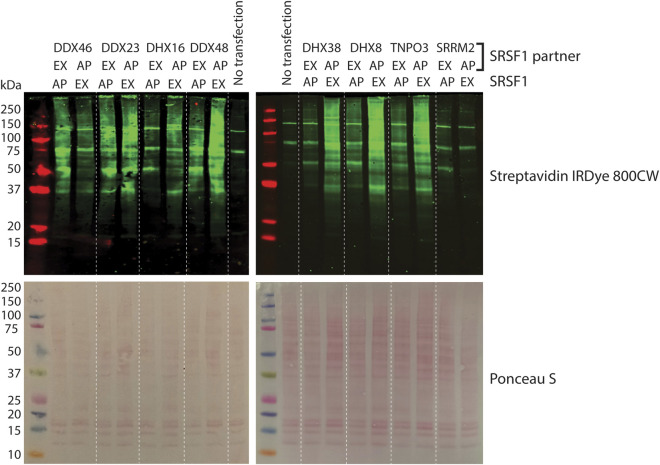
Effects of split-APEX combinations on the efficiency of biotinylation. AP or EX fragments of APEX2 fused to SRSF1 were co-transfected into HEK293T cells with EX or AP fragments fused to the helicases, TNPO3 or SRRM2. Cells were lysed in 8 M urea and the equivalent of 40 µg of total protein content was analysed by SDS-PAGE. Biotinylation was detected by Western blotting with dye-labelled streptavidin (upper panel) and equal loading and transfer were shown by staining the membrane with Ponceau S (lower panel).

To determine the optimal configuration of AP and EX fusions for each pair, both combinations were transfected into HEK293T cells and the levels of biotinylation assessed by Western blotting of the lysates. Control experiments with DDX46 and SRSF1 showed that there was no biotinylation if either the AP or EX fusions were transfected on their own (Supplementary Figure S2). When the AP and EX fusions were transfected together, successful biotinylation was seen (Supplementary Figure S2; Figure 1); the level was generally higher when SRSF1 was fused to the short EX fragment of APEX2, except when used in pairs with DHX38 and SRRM2, when SRSF1 was fused to the AP fragment. The optimal combinations were used in subsequent experiments. The functional activity of these SRSF1 fusions was tested by analysing their ability to enhance splicing of an intron in the 3′UTR of the SRSF1 pre-mRNA, an activity that mediates some autoregulation of SRSF1 expression (Ding et al., 2022; Lareau et al., 2007; Sun et al., 2010). All of the combinations being used resulted in increased levels of the spliced mRNA (Supplementary Figure S3).

Following scaled-up transfections, the levels of expression of the fusion protein compared with the endogenous protein were determined by Western blotting of the lysate (Supplementary Figure S4). In all cases, expression of the EX fragment fusion protein (usually SRSF1) was undetectable, and that of the AP fragment fusions was in most cases no higher than the level of the endogenous protein, with the exceptions of AP-DHX8 and AP-TNPO3. The very low levels of expression of the EX fusions reduce the probability that the APEX fragments would associate in the cell by collisions resulting from free diffusion rather than by being bound in close proximity. Biotinylated proteins were recovered from the lysates and analysed by MS/MS mass spectrometry. In addition, two further replicates were done for each combination, so altogether three independent experiments were analysed (Supplementary Figure S5). Every experiment included three negative controls, in which the cells had not been transfected but had been treated otherwise as the transfected cells. Scaffold software was used to produce a total spectral count for each protein identified, and this was used to estimate the relative yields of each protein in different samples (Old et al., 2005; Schmidt et al., 2014).

**FIGURE 2:**
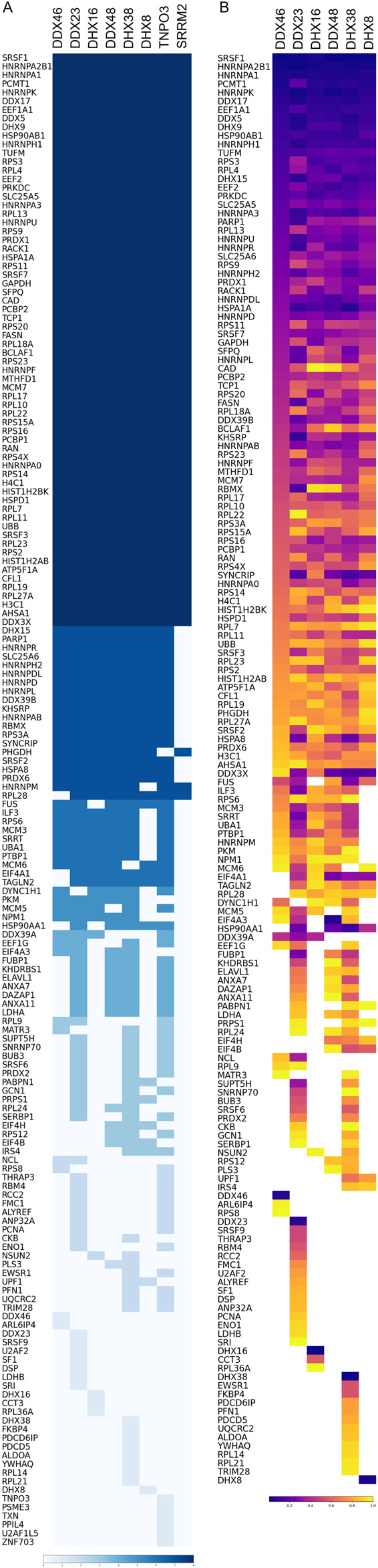
**(A)** List of proteins found with at least one combination of an SRSF1 fusion to AP or EX and a helicase, TNPO3 or SRRM2 fused to EX or AP. Each combination was tested in three separate experiments, and each dataset lists proteins found in at least two experiments. These proteins are arranged in blocks according to the number of datasets in which the proteins were found, indicated by the depth of colour of the block. **(B)** The proteins in the DDX46-SRSF1 dataset are listed in blocks according to the number of other helicase datasets in which they were found. Within each block the proteins are ranked according to the total 679 spectral counts of each protein (highest at the top; dark purple). The same proteins in the other datasets are coloured according to their ranking in the total spectral counts in that dataset.

**FIGURE 3:**
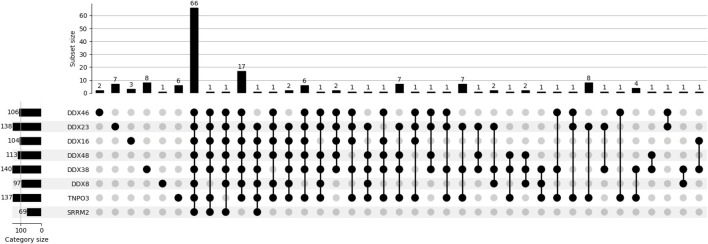
Similarities and differences among datasets. An UpSet plot shows the numbers of proteins found in each of the possible combinations of the datasets. Bar charts show the numbers of proteins in each dataset and the numbers found in any particular combination of datasets.

The lists of proteins identified in each condition contained only those proteins found in at least two replicates. In addition, the lists were edited to remove keratin, tubulin, actin and endogenously biotinyated proteins (Niers et al., 2011), which were the only proteins found in the negative control samples. The lists comprised 106, 138, 104, 113, 140, 97, 137 and 69 proteins respectively for the combinations of SRSF1 with the targets DDX46, DDX23, DHX16, DDX48, DHX38, DHX8, TNPO3 and SRRM2 (Figure 2; Supplementary Table S1). This compares with 917 proteins labelled by the full-length APEX fused to SRSF1, suggesting that the SRSF1-helicase combinations were either less efficient or labelled a restricted set of proteins. UpSet plots show the numbers of proteins common to two or more experiments (Figure 3). Strikingly, all the split-APEX experiments labelled a core set of 66 proteins, and the SRSF1-helicase combinations labelled an additional 18, producing 84 proteins that were common to all the SRSF1-helicase combinations (Figure 2; Supplementary Table S2).

To compare the sets of proteins, the proteins in each set were ranked according to their total spectral count. Both SRSF1 and the tagged helicase appeared in the top 10 hits of each SRSF1/helicase set, as might be expected. However, in most cases the helicases do not appear in the sets generated using other helicases (e.g., DDX46 appears only when it was tagged, and not in the sets from SRSF1/DDX23, etc.). The only exception is DDX48 (eIF4A3), which does appear in the SRSF1/DDX23 set. In contrast, all the helicases were recovered in the APEX2-SRSF1 set. One possible inference is that each helicase interacts with SRSF1 only when the other helicases are not in close proximity. TNPO3 was identified only in the SRSF1/TNPO3 experiment, where it was ranked only 37th, and it was not in the APEX2-SRSF1 set. SRRM2 did not appear in the SRRM2/SRSF1 experiment, and in the APEX2-SRSF1 set it was not ranked highly. Other helicases appear more abundantly: DDX5, DDX17 and DHX9 are abundant in all of the sets. The highest-ranking proteins generally are the hnRNPs: A1, A2B1, K and H1.

**FIGURE 4:**
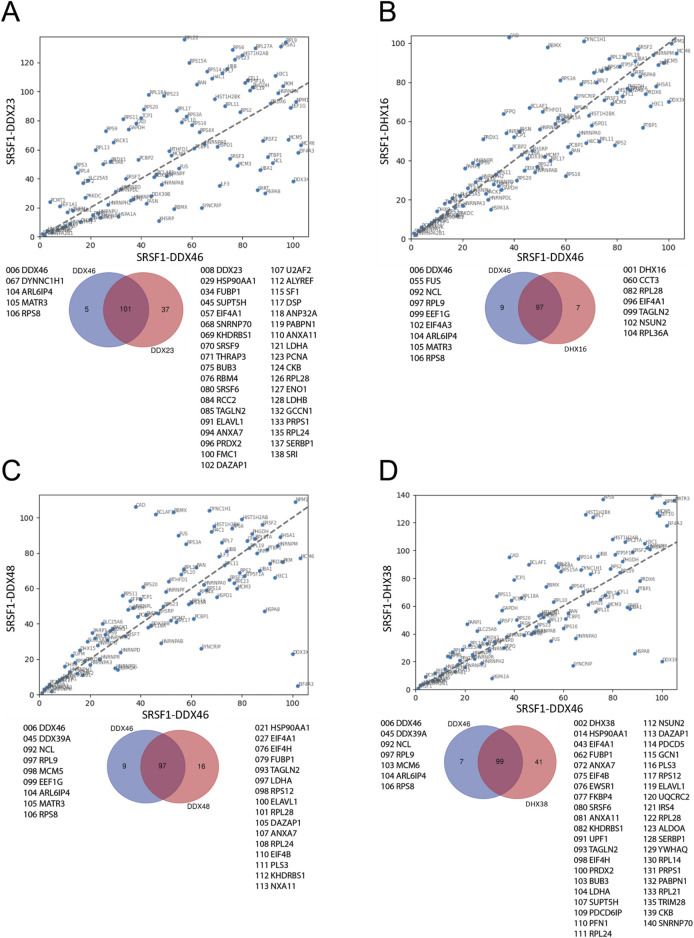
Comparisons between the ranking of proteins in different SRSF1-helicase datasets. The diagonal plots are based on the ranking of proteins in each of two datasets. Venn diagrams were used to identify the differences in the presence/absence of proteins between the datasets. The adjacent lists show the proteins that are unique to each member of the pair. Numbers in front of the proteins indicate the ranking position. **(A)** SRSF1+DDX46 vs. SRSF1+DDX23. **(B)** SRSF1+DDX46 vs. SRSF1+DHX16 comparison. **(C)** SRSF1+DDX46 vs. SRSF1+DDX48. **(D)** SRSF1+DDX46 vs. SRSF1+DHX38.

**FIGURE 5:**
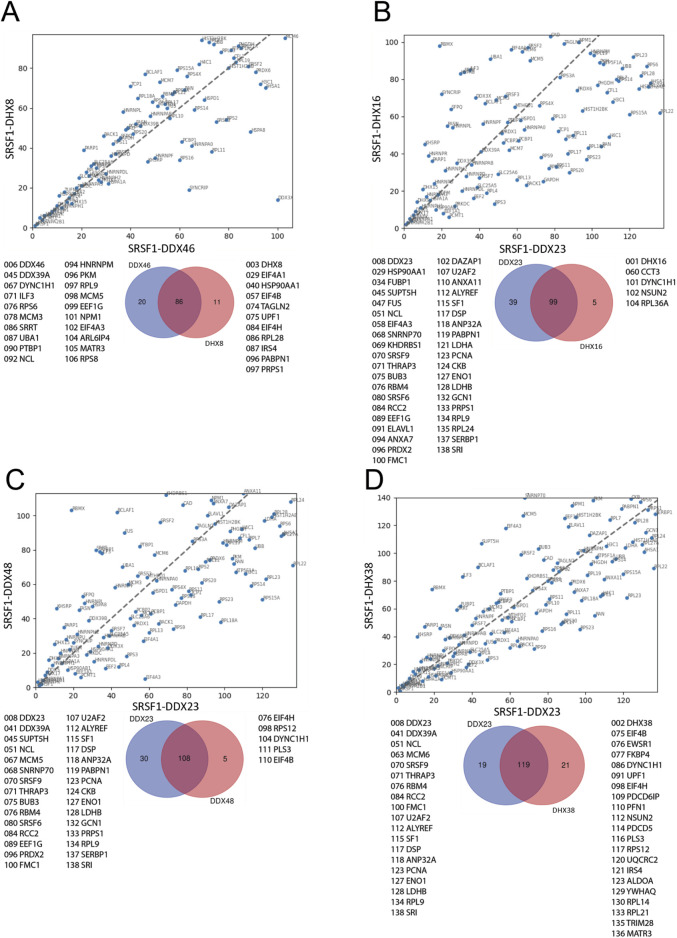
Comparisons between the ranking of proteins in different SRSF1-helicase datasets. The diagonal plots are based on the ranking of proteins in each of two datasets. Venn diagrams were used to identify the differences in the presence/absence of proteins between the datasets. The adjacent lists show the proteins that are unique to each member of the pair. Numbers in front of the proteins indicate the ranking position. **(A)** SRSF1+DDX46 vs. SRSF1+DHX8. **(B)** SRSF1+DDX23 vs. SRSF1+DHX16. **(C)** SRSF1+DX23 vs. SRSF1+DDX48. **(D)** SRSF1+DDX23 vs. SRSF1+DHX38.

**FIGURE 6:**
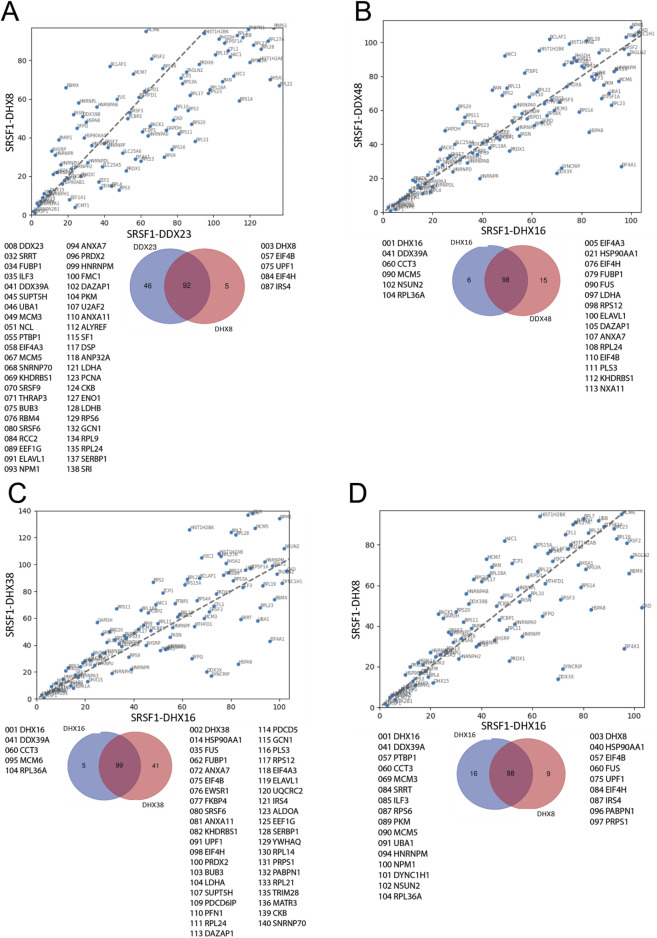
Comparisons between the ranking of proteins in different SRSF1-helicase datasets. The diagonal plots are based on the ranking of proteins in each of two datasets. Venn diagrams were used to identify the differences in the presence/absence of proteins between the datasets. The adjacent lists show the proteins that are unique to each member of the pair. Numbers in front of the proteins indicate the ranking position. **(A)** SRSF1+DDX23 vs. SRSF1+DHX8. **(B)** SRSF1+DHX16 vs. SRSF1+DDX48. **(C)** SRSF1+DHX16 vs. SRSF1+DHX38. **(D)** SRSF1+DHX16 vs. SRSF1+DHX8.

**FIGURE 7:**
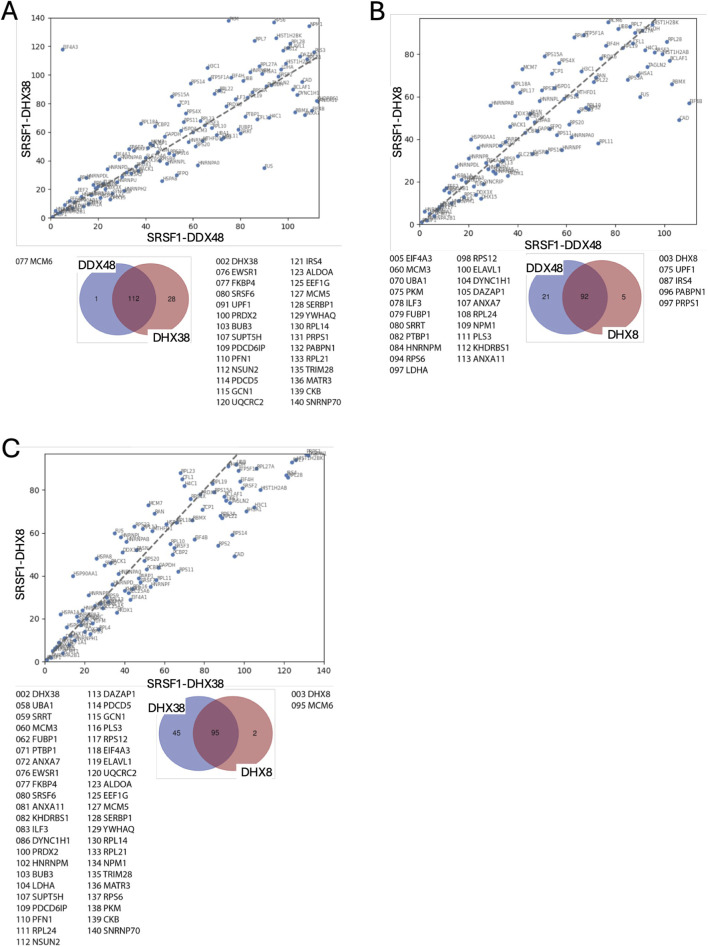
Comparisons between the ranking of proteins in different SRSF1-helicase datasets. The diagonal plots are based on the ranking of proteins in each of two datasets. Venn diagrams were used to identify the differences in the presence/absence of proteins between the datasets. The adjacent lists show the proteins that are unique to each member of the pair. Numbers in front of the proteins indicate the ranking position. **(A)** SRSF1+DDX48 vs. SRSF1+DHX38. **(B)** SRSF1+DDX48 vs. SRSF1+DHX8. **(C)** SRSF1+DHX38 vs. SRSF1+DHX8.

**FIGURE 8:**
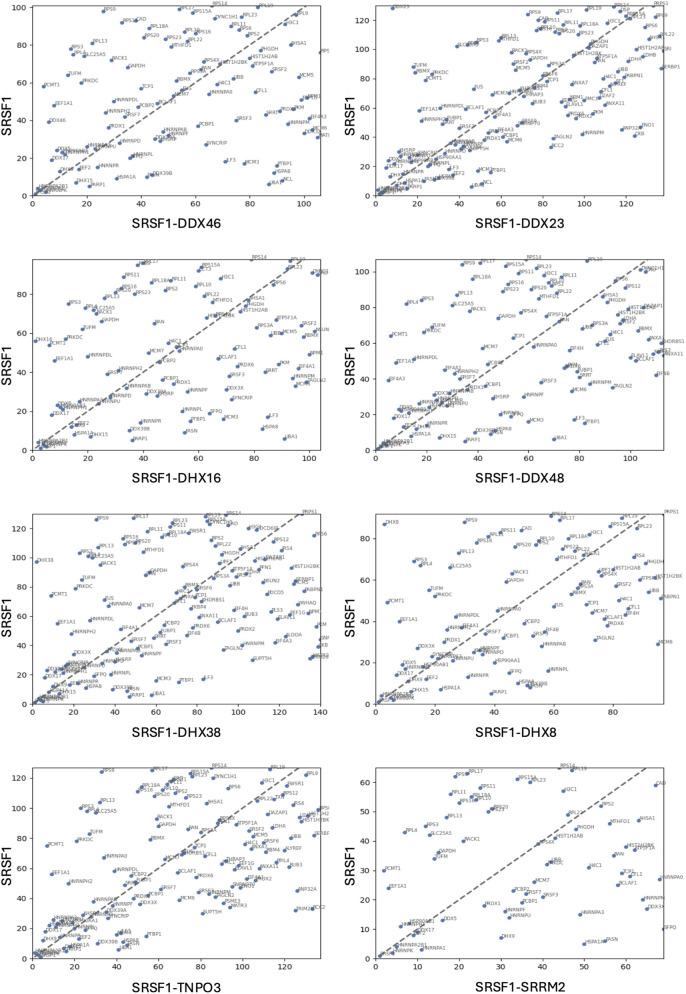
Diagonal plots comparing the rankings of proteins in common between the APEX2-SRSF1 and the split-APEX2 datasets. The ordinate shows proteins ranked in the APEX2-SRSF1 dataset, and the abscissa shows proteins ranked in the split-APEX2 dataset.

The ranked sets were compared pair-wise in diagonal plots to assess their overall similarity. Strikingly, the proteins in common between any two helicase-SRSF1 combinations showed clear convergence towards the line expected if the ranks were identical (Figures 4–7). To test whether rankings depended on the helicase partner or were merely the outcome of labelling directed by SRSF1, the common factor in all the tests, the ranked sets were plotted against the ranked set produced by APEX2-SRSF1 alone (Figure 8). Some of these plots showed no apparent correlation, apart from a possible cluster of highly abundant proteins near the origin (SRSF1, of course, and hnRNPs A1, A2B1, and K), such as the plots of APEX2-SRSF1 hits vs. combinations of SRSF1/DDX46, SRSF1/DDX48 and SRSF1/SRRM2.

**FIGURE 9:**
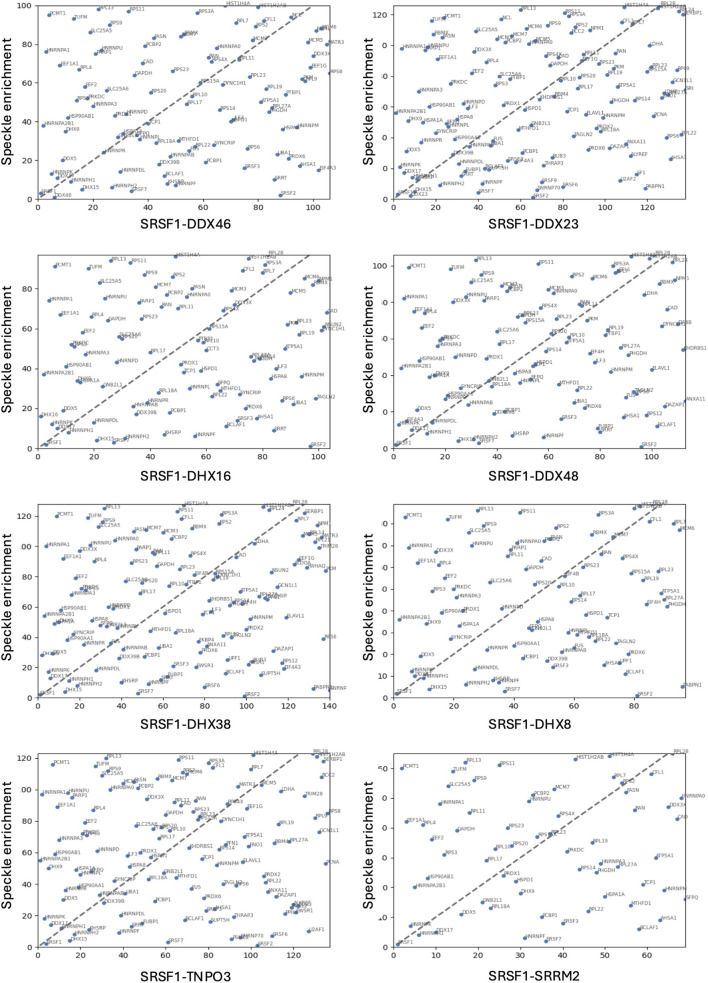
Diagonal plots comparing the rankings of proteins in common between a previously-reported APEX dataset from speckles (Dopie et al., 2020) and the split-APEX2 datasets.

To test further whether the results might be consequent upon sequestration into speckles by SRSF1, the lists were compared with previous data in which proteins enriched in speckles were determined by indirect peroxidase labelling (Dopie et al., 2020) (Figure 9). No correlation was observed. Even the SRSF1/SRRM2 combination did not label SRRM2 or SON, which are major speckle factors (Dopie et al., 2020; Ilik et al., 2020). Another possibility is that the results reflected the concentrations of proteins in the cells and that the limited numbers of proteins found simply reflected a limiting efficiency. Thus, the lists were compared with the results with previous measurements of protein abundance in HeLa cells (Hein et al., 2015), although these was not based on peroxidase labelling, but again no correlation was observed (Supplementary Figure S6). The results therefore suggest that the sites in the cell at which SRSF1 comes into contact with helicases, SRRM2 or TNPO3 are in some sense unique.

**FIGURE 10:**
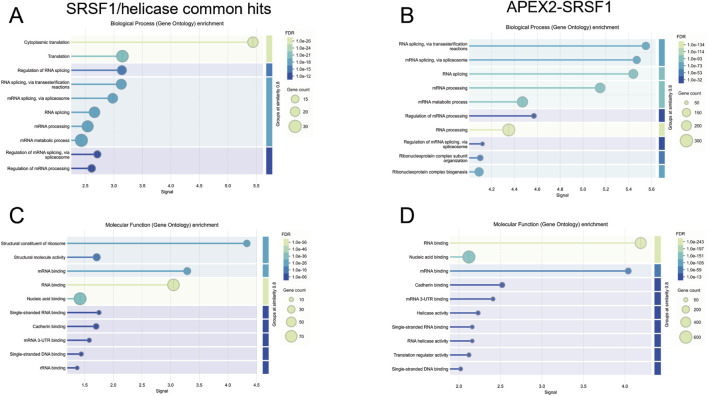
Gene ontology analyses of the proteins common to all SRSF1-helicase split-APEX2 datasets and proteins in the full-length APEX2-SRSF1 dataset. The String database was used for analysis of biological processes **(A,B)** and molecular function **(C,D)**. Panels A and C show the proteins common to all the SRSF1-helicase datasets, while B and D include all the proteins in the APEX2-SRSF1 dataset.

**FIGURE 11:**
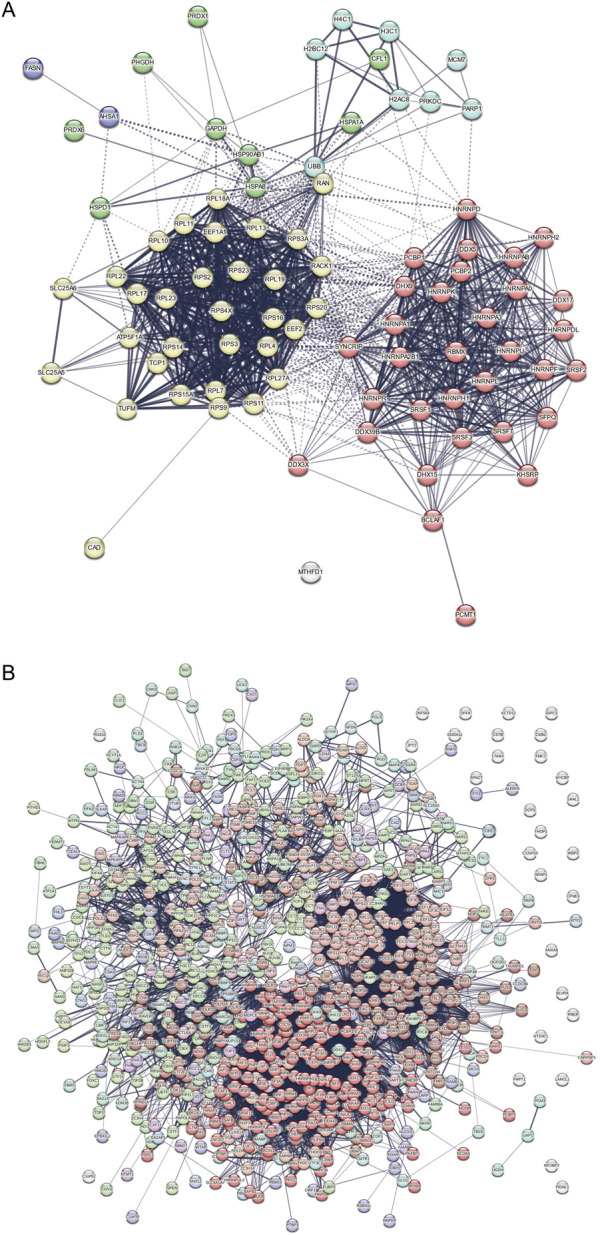
String protein networks. **(A)** Network for the proteins common to all the SRSF1-helicase split-APEX2 datasets. Edges indicate both functional and physical protein associations. The line thickness indicates the strength of data support. The proteins were clustered using MCL clustering and the edges between clusters are represented by dotted lines. **(B)** Network for the full-length APEX2-SRSF1 dataset.

Among the 84 proteins common to all the SRSF1-helicase pairs, gene ontology analysis based on biological process revealed a very marked enrichment of terms related to translation and splicing (Figure 10A; Supplementary Table S2), whereas the 917 proteins labelled with full-length APEX2 fused to SRSF1 were predominantly assigned to splicing or RNA processing (Figure 10B). Likewise, an analysis of molecular function showed enrichment for ribosome constituents and mRNA binding (Figure 10C) and ther APEX2-SRSF1 hits were predominantly involved in RNA binding (Figure 10D). This contrast is revealed very clearly in a bi-lobed STRING plot of protein associations for the split-APEX hits (Figure 11A) in comparison with a single network for APEX2-SRSF1 (Figure 11B). It is possible that the association of SRSF1 with the helicases, SRRM2 or TNPO3 occurs during ribosomal biogenesis as well as splicing.

## Discussion

The intentions of these experiments were to establish whether all the splicing helicases are at any point sufficiently close to SRSF1 for the two portions of the split APEX2 enzyme to coalesce into a single functional enzyme and reconstitute peroxidase activity, thereby enabling information to be gained about the local environment when they interact directly. The numbers of proteins detected with each combination of SRSF1 and helicase are all quite similar, which suggests that SRSF1 associates as much in the cell with the other helicases as it does with DDX23 or TNPO3, interactions that have been confirmed previously (Maertens et al., 2014; Segovia et al., 2024). However, if the interactions took place at discrete points in the splicing pathway, then spliceosomal components would be disproportionately enriched compared with other nuclear proteins and, moreover, those components that are characteristic of the stage in splicing at which the helicase was associated would be enriched in the appropriate SRSF1/helicase experiment and not in the others. The U1 snRNP 70K protein, which would be expected to be present with DDX46 and DDX23, appears in the middle of the SRSF1/DDX23 list and elsewhere only as the lowest-ranked entry in the DHX38 list. Few of the unique hits had any links to splicing. The main exception was the SRSF1-DDX23 pair, which revealed two early stage proteins, U2AF2 and SF1, and SRSF9. Neither of the early stage proteins would be expected to be present when DDX23 enters with the tri-snRNP into association with complex A to form the pre-B complex (Charenton et al., 2019; Zhang Z. et al., 2024), since they would be displaced when complex A forms (Chen et al., 2017; Fleckner et al., 1997; Martinez-Lumbreras et al., 2024; Tholen et al., 2022), but there are other reports suggesting some continuing engagement in complex A (Agafonov et al., 2011). The other exception was with the SRSF1-DDX46 pair, which produced ARL6IP4. This protein, also known as SRp37, is an SR-like protein that has been located in nuclear speckles and nucleoli and has been shown to affect alternative splicing (Ouyang, 2009). Nothing is known of the mechanisms by which it affects splicing, but involvement at an early stage when DDX46 is present is possible. The role of this protein in splicing reactions would seem to be worth further investigation. However, we did not identify other examples of potential stage-specific spliceosomal proteins that are enriched in just one list, and the lists are dominated in the top dozen ranks by factors regulating splicing, which are generally present at higher concentrations than the spliceosomal factors (Hein et al., 2015).

These results raise questions as to the nature of the compartments, virtual or actual, in the cell in which SRSF1 interacts with helicases, SRRM2 and TNPO3. Since there is no substantial similarity in ranking to that seen with SRSF1 alone, factors such as the abundance of a protein or the presence of electrophilic amino acids do not seem to be the sole determinants of the more restricted outcomes seen with split-APEX. It will be useful to determine the location within the cell of the fusion proteins, although the absence of highly abundant cytoplasmic proteins favours the expected nuclear location. A potential limitation to these experiments is that the low concentrations of the EX fusions might limit the ability to detect all but the tightest interactions or interactions at those sites where the fusion protein is most concentrated, even if the interaction normally takes place and is functional but transient at other sites in the cell. A major drawback to experiments with APEX, split or entire, is the high mobility of the biotin-phenoxy radical. The labelling efficiency declines with distance and time from the source, but labelling is detected out to 20–270 nm from the source (Hung et al., 2016; Oakley et al., 2022). Improving the resolution of labelling might be achieved by less stable radicals, bulkier radicals that diffuse more slowly or the use of the expansion methods used in microscopy. Carbene-based radicals have already been shown to enable a higher resolution (Bartholow et al., 2024; Geri et al., 2020; Oakley et al., 2022).

A striking feature of our split-APEX results is the high proportion of labelled proteins that are involved in translation or ribosomal biogenesis. STRING plots show this very clearly: the majority of the split-APEX hits belong to either the splicing or translation networks, whereas the APEX2-SRSF1 hits include a much smaller proportion of proteins linked to translation and they are subsumed into the pattern of a single extensive network (Figure 11). It is unlikely that the helicases, SRRM2 and TNPO3 are recruited by SRSF1 to sites where they would not normally be located as a result of interactions between the AP and EX domains, since the pattern of proteins labelled is so different from that of SRSF1 alone. Instead, our results raise the possibility that these proteins, together with SRSF1, have roles in translation or ribosomal biogenesis. There is already evidence that DDX46, DDX23, DDX48, DHX8 and one of the abundant common hits, DDX5, have some involvement in ribosomal biogenesis (Martin et al., 2013; Tafforeau et al., 2013), and many splicing factors including SRSF1 have been found in the nucleolus (Andersen et al., 2005). The yeast orthologue of SRSF1, Npl3, has a role in exporting the large pre-ribosomal subunit (Hackmann et al., 2011). The binding of SRSF1 to RNA is in competition with the folding of the RNA into G-quadruplexes (Smith et al., 2014), and it has been shown recently that it promotes the unfolding of quadruplexes (De Silva et al., 2024). It is therefore not beyond the bounds of possibility that SRSF1 and the helicases cooperate in the remodelling of rRNA during ribosomal biogenesis to enable the formation of long-range secondary structures or to remove snoRNAs (Rodgers and Woodson, 2021). There is at present still some uncertainty regarding the number and roles of helicases involved in rRNA maturation (Mitterer and Pertschy, 2022).

An alternative possibility is that SRSF1 and the helicases might be involved in the activities of long non-coding (lnc) RNA transcribed by RNA polymerases I or II in the nucleolus and localized either in the nucleolus or the perinucleolar compartment (Dogra and Kriwacki, 2025; Feng and Manley, 2022). Of particular interest is the exemplar PNCTR, a perinucleolar lncRNA that regulates pre-mRNA splicing by sequestering polypyrimidine tract-binding protein, (PTBP1) (Yap et al., 2018) and was found by APEX2 labelling to be in close proximity to a number of pre-mRNA splicing factors (Yap et al., 2022). Significantly, one of these is SRSF1 (Yap et al., 2022). Thus, another possibility is that SRSF1 and the protein partners we tested might be involved in the organisation and modelling of nucleolar or perinucleolar RNA to control the level of binding of splicing factors by regulatory RNA. A prerequisite for further research would be to identify the sites within the nucleus at which SRSF1 and the tested proteins are colocalized and to test whether pre-rRNA processing, lncRNA abundance or the localization of splicing factors in the nucleolar regions are affected by knockdown or mutations that affect the interactions of SRSF1 with the helicases.

## Materials and methods

### Cloning

The human DDX46, DHX16, DDX48, DHX38 and DHX8 genes were bought from Insight Biotechnology UK (RC202320, RC202912, SC114843, RC200044, SC324113). The human TNPO3 gene was bought from Sino Biological (HG18667-U). The human SRRM2 gene was a kind gift from Wesley Sundquist & Katie Ullman (Addgene plasmid # 174089). The AP and EX fragments were kind gifts from Alice Ting (Addgene plasmid # 120912, plasmid #120913) (Han et al., 2019). The pEGFP-C1 vector was used as a backbone to generate constructs encoding for AP- and EX-fused DDX46, DDX23, DHX16, DDX48, DHX38, DHX8, TNPO3 and SRRM2 (Supplementary Figure 1). The cloning was performed using In-Fusion seamless cloning (Takara) or NEBuilder HiFi DNA Assembly (New England Biolabs) according to the manufacturers’ instructions. All constructs were verified using Nanopore DNA sequencing (Source Bioscience, UK).

### Mammalian cell culture and transfections

HEK293T cells from ATCC (passages <20) were cultured in complete growth media consisting of 90% DMEM, GlutaMAX (Dulbecco’s Modified Eagle medium, Gibco) supplemented with 10% w/v FBS (Fetal Bovine Serum, Gibco) at 37 °C, under 5% CO_2_. Cells were transfected at 70%–80% confluence using jetPrime (Polyplus), with 10 μL jetPrime reagent, 500 μL jetPrime Buffer and 5 μg total DNA (AP-fusion: 2 μg, EX-fusion: 3 μg) per 9 × 10^6^ cells for 4 h, after which transfection media was replaced with fresh growth media. The cells were tested negative for *mycoplasma*.

### Biotin-phenol proximity labelling

HEK293T cells were transiently transfected with the plasmids encoding for the split APEX2 pairs. Labelling was done with the concentrations of biotin tyramide and hydrogen peroxide described in previous split-APEX experiments (Han et al., 2019). 24 h after the transfection, the growth media was changed with fresh growth media containing 500 μΜ Biotin tyramide, that was sonicated for at least 30 min. Cells were incubated at 37 °C, under 5% CO_2_ for 30 min, prior to labelling initiation with the addition of H_2_O_2_ (1 mM final concentration in PBS) and gentle agitation. The labelling reaction was quenched after 1 min with the removal of the growth media and the addition of Quenching solution (10 mM Sodium Ascorbate, 5 mM Trolox, 10 mM sodium azide in PBS). The cells were washed twice with the Quenching solution, before being harvested in Quenching solution by centrifugation at 1000 × g for 5 min.

### Western blotting

A small proportion of a cell pellet from the biotin-phenol labelling was used for Western blotting. Cells were incubated at 90 °C, for 10 min, in the presence of 8 M Urea and then resolved on 4%–12% v/v SDS-polyacrylamide gels (NuPAGE Invitrogen). The gels were transferred to nitrocellulose membranes, stained with Ponceau S (0.1% w/v Ponceau S, 5% v/v acetic acid) and blocked at 4 °C, overnight with SuperBlock T20 (PBS) Blocking Buffer (Fisher Scientific). Blots aiming at the detection of biotinylated proteins were immersed at room temperature, for 1 h in IRDye 800CW Streptavidin (Li-Cor, 926–32230, 1:5000 dilution), rinsed three times with Blocking buffer 10 min each time and visualised using Odyssey Imager (Li-Cor). Blots aiming at the detection of the AP/EX-fused pairs were immersed at room temperature, for 1 h in rabbit anti-DDX46 (Insight Biotechnology, GTX115308, 1:1000 dilution)/rabbit anti-DDX23 (Insight Biotechnology, GTX115234, 1:1000 dilution)/rabbit anti-DHX16 (Insight Biotechnology, GTX115088, 1:1000 dilution)/rabbit anti-DDX48 (Sino Biological, 200,323-T36, 1:500 dilution)/rabbit anti-DHX38 (Proteintech, 10098-2-AP, 1:1000 dilution)/rabbit anti-DHX8 (Stratech Scientific, C14659-ABT, 1:500 dilution)/rabbit anti-TNPO3 (Bio-Techne, NBP3-15908, 1:1000 dilution)/mouse anti-SRSF1 (gift from A.R. Krainer, CSH laboratory, 1:1000 dilution). The blots were rinsed with Blocking buffer three times for 10 min each time, before being immersed at room temperature, for 1 h in IRDye 680RD Goat anti-rabbit IgG secondary antibody (Li-Cor, 926–68071, 1:1000 dilution) or IRDye 800CW Goat anti-mouse IgG secondary antibody (Li-Cor, 926–32210, 1:1000 dilution). Then, blots were rinsed with Blocking buffer three times for 10 min each time and visualised using Odyssey Imager (Li-Cor).

### Sample preparation for mass spectroscopy

∼30 × 10^6^ cells were used for each sample. Cells were lysed in lysis buffer comprised of 50 mM Tris-HCl pH 7.5, 0.5 M NaCl, 0.4% w/v SDS, 1 mM EDTA, 1 mM DTT, 2% v/v TritonX-100, Roche Complete Protease Inhibitor Cocktail mix using an ultrasonic disintegrator. The amount of protein content in each sample was assessed in triplicate using a nanodrop spectrophotometer and normalised amount to the sample with the lowest protein content was loaded on pre-washed High capacity Neutravidin resin (Thermo Fisher Scientific, #29204). The lysates were incubated with the resin at 4 °C, overnight with rotation. The resin was washed and pelleted by centrifugation at 1500 *g*, 4 °C, 5 min three times with each of the following buffers wash 1 (2% w/v SDS), wash 2 (50 mM Tris-HCl pH 7.5, 0.5 M NaCl, 1 mM EDTA, 1% v/v TritonX-100, 0.1% w/v deoxycholate), wash 3 (10 mM Tris-HCl pH 8.0, 250 mM LiCl, 1 mM EDTA, 0.5% w/v deoxycholate) and wash 4 (50 mM Tris-HCl pH 8.0), wash 5 (100 mM Triethylammonium bicarbonate (TEAB)). Then, the samples were reduced and alkylated with the addition of buffer containing 100 mM TEAB, 40 mM Iodoacetamide (IAA), 10 mM DTT at 70 °C for 20 min (gentle agitation) prior to cooling down to 37 °C and overnight trypsinisation with 1 μg trypsin (Thermo Scientific) at 37 °C, overnight (gentle agitation). The next day the samples were acidified using Trifluoroacetic acid (TFA) (3<pH < 4). The Mass Spectroscopy initial analysis was performed at Warwick Scientific Services, University of Warwick.

### RNA extraction - Reverse transcription–PCR

Total RNA was extracted using the Qiagen RNeasy Mini kit, with the addition of the on-column DNase treatment (Qiagen) to remove any residual DNA. PrimeScript RT master Mix was used for the cDNA synthesis (Takara). OneTaq 2x Master Mix was used for the PCR reactions (NEB). In all cases, the manufacturers’ protocols were followed. Bands from agarose gels were quantified using Fiji (Schindelin et al., 2012).

### Gene ontology analysis

Gene Ontology (GO) analysis was performed using ShinyGO 0.80 (Ge et al., 2020) and the String database (Szklarczyk et al., 2023). KEGG pathway database was also used (Kanehisa et al., 2023; Kanehisa & Goto, 2000). Circos plots were generated with Circos software (Krzywinski et al., 2009) and Metascape (Zhou et al., 2019). Venn/Euler diagrams were generated using the Venn diagram tool from Gent University (https://bioinformatics.psb.ugent.be/webtools/Venn).

## Data Availability

The Scaffold files have been published on Figshare: https://doi.org/10.25392/leicester.data.30217723.
