# Supplementary material for: Split-APEX implicates splicing factor SRSF1 and splicing helicases in ribosomal biogenesis
Source: Front Mol Biosci. 2025 Dec 19;12:1714378. doi: 10.3389/fmolb.2025.1714378 (PMC12757297; doi:10.3389/fmolb.2025.1714378)
Supplement: Supplementary file 1 [file Supplementaryfile1.pdf]

Supplementary Materials

Supplementary Table 1. Datasets of proteins appearing in two or more replicates of split-APEX2 experiments with SRSF1 and the helicases, TNPO3 or SRRM2, and APEX-SRSF1

DDX46/Prp5	DDX23/Prp28	DHX16/Prp2	DDX48/eIF4A3	DHX38/Prp16	DHX8/Prp22	TNPO3	SRRM2	APEX-SRSF1
SRSF1	HNRNPA2B1	DHX16	SRSF1	SRSF1	SRSF1	HNRNPA2B1	SRSF1	SRSF1
HNRNPA2B1	SRSF1	HNRNPA2B1	HNRNPK	DHX38	HNRNPA2B1	HNRNPA1	PCMT1	HNRNPK
HNRNPA1	HNRNPK	SRSF1	HNRNPA2B1	HNRNPA2B1	DHX8	HNRNPK	EEF1A1	HNRNPA1
PCMT1	HNRNPA1	HNRNPA1	HNRNPA1	HNRNPA1	PCMT1	SRSF1	HNRNPK	HNRNPA2B1
HNRNPK	DDX17	HNRNPK	EIF4A3	HNRNPK	HNRNPA1	DHX9	HNRNPA2B1	PARP1
DDX46	DDX5	PCMT1	PCMT1	DDX17	HNRNPK	DDX17	HNRNPH1	UBA1
DDX17	DHX9	DDX17	DDX17	DDX5	DDX17	PCMT1	RPL4	DHX15
EEF1A1	DDX23	EEF1A1	EEF1A1	HSPA1A	EEF1A1	HSP90AB1	HSP90AB1	NCL
DDX5	HNRNPH1	DDX5	DDX5	PCMT1	DDX5	EEF1A1	EEF2	HSPA1A
DHX9	DHX15	HSP90AB1	HSP90AB1	DHX9	HNRNPH1	HNRNPA3	DDX17	SNRNP200
HSP90AB1	KHSRP	HNRNPH1	EEF2	HSP90AB1	DHX9	HNRNPH1	HNRNPA1	XRCC6
HNRNPH1	HNRNPA3	PRKDC	RPL4	EEF1A1	DHX15	DDX5	RPS3	FASN
TUFM	HNRNPR	RPS3	HNRNPH1	DHX15	RPS3	HNRNPR	SLC25A5	SF3B1
RPS3	HSPA1A	DHX9	HSPA1A	HSP90AA1	DDX3X	HNRNPU	TUFM	EFTUD2
RPL4	PARP1	HSPA1A	HNRNPDL	HNRNPH1	RPL4	DHX15	GAPDH	DDX39B
DHX15	HNRNPU	EEF2	DHX9	HNRNPA3	HSP90AB1	PARP1	DDX5	HSPA8
EEF2	HSP90AB1	HNRNPA3	HNRNPA3	SYNCRIP	EEF2	HNRNPH2	RPL13	DHX9
PRKDC	TUFM	TUFM	RPS3	EEF2	TUFM	HSPA1A	RPL11	EEF2
SLC25A5	RBMX	RPL4	PRKDC	HNRNPH2	SYNCRIP	HSP90AA1	RPS9	HNRNPR
HNRNPA3	SYNCRIP	HNRNPDL	HNRNPR	DDX3X	PRKDC	HNRNPL	RPS16	PTBP1
PARP1	EEF1A1	DHX15	HSP90AA1	PRKDC	HNRNPA3	PRKDC	RACK1	MCM3
RPL13	HNRNPH2	SLC25A5	TUFM	HNRNPR	HSPA1A	EEF2	RPL17	ILF3
HNRNPU	FASN	RACK1	DDX3X	RPS3	PRDX1	RPS3	RPL18A	TKT
HNRNPR	PCMT1	HNRNPU	HNRNPD	TUFM	HNRNPH2	KHSRP	RPL10	DDX17
SLC25A6	SFPQ	GAPDH	DHX15	HNRNPU	SLC25A5	SFPQ	RPS11	SFPQ
RPS9	HNRNPL	RPL13	SYNCRIP	HSPA8	HNRNPU	RPL4	PRDX1	HNRNPL
HNRNPH2	PRKDC	SRSF7	EIF4A1	RPL4	HNRNPDL	TUFM	RPS23	HNRNPH1
PRDX1	DDX39B	HNRNPD	HNRNPU	HNRNPDL	RPL13	HNRNPD	RPS20	HSP90AB1
RACK1	HSP90AA1	SLC25A6	HNRNPAB	SLC25A5	EIF4A1	HNRNPAB	HSPD1	DDX5
HNRNPDL	HSPA8	RPS20	SLC25A5	SFPQ	RPS9	SLC25A5	DHX9	HSP90AA1
HSPA1A	HNRNPDL	HNRNPH2	HNRNPH2	RPS9	HNRNPR	DDX39B	HNRNPF	HNRNPU
HNRNPD	SRRT	RPS16	RPL13	RPL13	SLC25A6	RPL13	HNRNPU	HNRNPA3
RPS11	HNRNPD	RPS11	SRSF7	RACK1	KHSRP	RPS9	PCBP2	SYNCRIP
SRSF7	FUBP1	HNRNPAB	RPS9	HNRNPD	RPS16	HNRNPA0	RPS15A	HNRNPD
GAPDH	ILF3	DDX39B	PARP1	FUS	HNRNPF	DDX39A	PCBP1	KHSRP
SFPQ	HNRNPAB	PARP1	PRDX1	PRDX1	HNRNPD	SYNCRIP	SRSF7	SUPT5H
HNRNPL	EEF2	RPS23	RACK1	HNRNPA0	SRSF7	TNPO3	RPL23	DDX39A
CAD	DDX3X	RPS9	RPL18A	HNRNPL	RPL11	HNRNPF	MCM7	GMPS
PCBP2	SLC25A5	HNRNPR	DDX39B	DDX39B	PARP1	HNRNPDL	RPS4X	VCP
TCP1	SRSF7	RPL17	SLC25A6	KHSRP	HSP90AA1	MCM3	SRSF3	TRIM28
RPS20	DDX39A	DDX39A	RPL17	HNRNPAB	HNRNPA0	UBA1	PRKDC	RCC2
FASN	HNRNPF	MCM7	MCM7	SLC25A6	RPS11	ILF3	UBB	PRPF8
RPL18A	BCLAF1	RPL18A	PCBP1	EIF4A1	PCBP1	HSPA8	HIST1H2AB	MATR3
DDX39B	RPL4	RAN	PCBP2	RPS16	GAPDH	SRRT	RPS14	MCM2
DDX39A	SUPT5H	KHSRP	FASN	RPS23	SFPQ	FASN	H3C1	HSPA4
BCLAF1	UBA1	PCBP2	KHSRP	FASN	RACK1	SLC25A6	RPL22	HNRNPF
KHSRP	FUS	PCBP1	HSPA8	PARP1	RPS20	PCBP2	RPL19	SRRM2
HNRNPAB	RPS3	RPS2	GAPDH	SRSF7	HSPA8	PRDX1	PHGDH	DDX3X
RPS23	MCM3	H4C1	HNRNPL	RPL17	CAD	FUBP1	HNRNPA3	PSME3
HNRNPF	SLC25A6	RPL11	RPS23	RPS20	PCBP2	RPS16	HSPA1A	HNRNPAB

MTHFD1	NCL	PRDX1	RPS15A	PCBP1	DDX39B	DDX3X	H4C1	DDX21
MCM7	PRDX1	TCP1	RPS16	MCM7	FASN	RPL18A	RPL7	SSRP1
RBMX	PCBP2	HNRNPA0	SFPQ	HNRNPF	SRSF3	HSPD1	RPL27A	MCM6
RPL17	SRSF3	HNRNPL	TCP1	MTHFD1	RPS2	PTBP1	RPS2	TMPO
FUS	PTBP1	FASN	HSPD1	RAN	RPL10	PCBP1	FASN	PRDX1
RPL10	MCM7	HNRNPF	RPS11	RPL11	HNRNPAB	RBMX	MTHFD1	TOP1
RPL22	EIF4A1	PTBP1	RPS4X	GAPDH	EIF4B	RPL17	RAN	TAGLN2
RPS3A	EIF4A3	HSPD1	HNRNPF	UBA1	HNRNPL	RPS20	BCLAF1	EIF5A
RPS15A	MTHFD1	RPL10	RPS14	SRRT	RPS14	RACK1	TCP1	PCBP1
RPS16	RPL13	CCT3	MCM3	MCM3	FUS	GAPDH	HIST1H2BK	CKB
PCBP1	PCBP1	RPS15A	RPS20	HSPD1	MTHFD1	SRSF7	CFL1	HNRNPM
RAN	HSPD1	RPL22	HNRNPA0	FUBP1	RPL17	RPL10	ATP5F1A	DDX46
RPS4X	MCM6	HIST1H2BK	RPL23	RPL10	RPS23	MCM7	AHSA1	MCMBP
SYNCRIP	HNRNPA0	SFPQ	MTHFD1	PCBP2	HSPD1	RPL11	HNRNPM	SRSF3
HNRNPA0	RACK1	MTHFD1	SRSF3	SRSF3	RPL18A	MTHFD1	DDX3X	GTF2I
RPS14	SRSF2	RPS4X	H3C1	RPL18A	RBMX	RPS11	RPL28	EIF4A3
DYNC1H1	MCM5	BCLAF1	ATP5F1A	RPS11	RPL22	CAD	CAD	PFAS
H4C1	SNRNP70	H3C1	RPL10	RPL23	RPS3A	RPS2	HNRNPA0	ANP32A
HIST1H2BK	KHDRBS1	MCM3	RPS2	CFL1	RAN	BCLAF1	SFPQ	SRSF7
HSPD1	SRSF9	DDX3X	UBA1	H4C1	AHSA1	MCM6		SUPT16H
ILF3	THRAP3	SRSF3	RPL22	PTBP1	TCP1	KHDRBS1		ENO1
RPL7	RPS4X	SYNCRIP	RAN	ANXA7	H3C1	FUS		SNW1
RPL11	RPS9	CFL1	RPL11	RPS4X	MCM7	RPS23		HTATSF1
UBB	GAPDH	PRDX6	PRDX6	RBMX	TAGLN2	RAN		SNRNP70
SRSF3	BUB3	HIST1H2AB	PKM	EIF4B	UPF1	RPS15A		HSPH1
RPS6	RBM4	RPL27A	EIF4H	EWSR1	RPS4X	RPL23		SRRT
RPL23	RPS16	PHGDH	MCM6	FKBP4	BCLAF1	TCP1		CCT2
MCM3	CAD	AHSA1	ILF3	PRDX6	PRDX6	PRDX6		CDC5L
RPS2	RPL10	RPS14	FUBP1	TCP1	RPS15A	SRSF3		DDB1
HIST1H2AB	SRSF6	RPL7	SRRT	SRSF6	HIST1H2AB	RPS3A		SRSF9
ATP5F1A	RPS11	RPS3A	UBB	ANXA11	SRSF2	SUPT5H		ALDOA
CFL1	TCP1	RPL28	PTBP1	KHDRBS1	H4C1	CFL1		RPA1
RPL19	RPS3A	HSPA8	RPL19	ILF3	RPL19	AHSA1		SF3B2
PHGDH	RCC2	SRRT	HNRNPM	RPL19	EIF4H	HNRNPM		EIF4B
RPL27A	TAGLN2	ILF3	RPS3A	RPS15A	CFL1	RPS14		HNRNPH2
SRRT	RPS2	UBB	RPL7	DYNC1H1	RPL28	DYNC1H1		FUBP1
UBA1	RPL17	RPS6	CFL1	RPS2	IRS4	TAGLN2		NASP
SRSF2	RPS20	ATP5F1A	RPL27A	RPS3A	RPL23	PFN1		PRDX2
HSPA8	EEF1G	PKM	AHSA1	RPL22	ATP5F1A	RPS4X		EIF4A1
PTBP1	RPL11	MCM5	FUS	BCLAF1	RPL27A	FMC1		PRDX6
PRDX6	ELAVL1	UBA1	PHGDH	UPF1	PHGDH	PSME3		PKM
NCL	PRDX6	RPL23	H4C1	PHGDH	UBB	THRAP3		MCM4
H3C1	NPM1	RPL19	TAGLN2	TAGLN2	RPL7	RPS6		PCBP2
HNRNPM	ANXA7	HNRNPM	RPS6	RPS14	HIST1H2BK	MATR3		EEF1A1
AHSA1	HIST1H2BK	MCM6	HIST1H2BK	CAD	MCM6	SNRNP70		HDGF
PKM	PRDX2	EIF4A1	SRSF2	UBB	PABPN1	ELAVL1		CTTN
RPL9	RPS23	SRSF2	LDHA	ATP5F1A	PRPS1	ATP5F1A		NONO
MCM5	RPL18A	RBMX	RPS12	EIF4H		EEF1G		SF3A1
EEF1G	HNRNPM	TAGLN2	HIST1H2AB	SRSF2		ENO1		BCLAF1
DDX3X	FMC1	NPM1	ELAVL1	PRDX2		RPL7		SUPT6H
NPM1	RPL19	DYNC1H1	RPL28	AHSA1		H4C1		DNMT1
EIF4A3	DAZAP1	NSUN2	BCLAF1	HNRNPM		EIF4A1		HNRNPDL
MCM6	PHGDH	CAD	RBMX	BUB3		SRSF2		HNRNPC
ARL6IP4	PKM	RPL36A	DYNC1H1	LDHA		ANXA7		USP7
MATR3	RAN		DAZAP1	H3C1		MCM5		ADAR
RPS8	ATP5F1A		CAD	RPL27A		PRDX2		HCFC1
	U2AF2		ANXA7	SUPT5H		RPL22		ELAVL1
	CFL1		RPL24	HIST1H2AB		ANXA11		ANXA11

	H4C1		NPM1	PDCD6IP		H3C1		BUB3
	ANXA11		EIF4B	PFN1		SRSF6		EEF1G
	H3C1		PLS3	RPL24		RBM4		CMTR1
	ALYREF		KHDRBS1	NSUN2		DAZAP1		HSPA6
	RPL7		ANXA11	DAZAP1		RPL19		H2AFX
	RPS14			PDCD5		LDHA		FMC1
	SF1			GCN1		TXN		CLTC
	UBB			PLS3		PPIL4		NPM1
	DSP			RPS12		UQCRC2		PLS3
	ANP32A			EIF4A3		RPL27A		SLC4A1AP
	PABPN1			ELAVL1		RPS12		SART1
	RPS15A			UQCRC2		ALYREF		ILF2
	LDHA			IRS4		EWSR1		BOP1
	RPL23			RPL28		BUB3		EIF4H
	PCNA			ALDOA		UBB		FKBP4
	CKB			RPL7		IRS4		U2AF2
	HIST1H2AB			EEF1G		TRIM28		PPIL4
	RPL28			HIST1H2BK		ANP32A		THRAP3
	ENO1			MCM5		U2AF1		CSDE1
	LDHB			SERBP1		HIST1H2BK		MCM7
	RPS6			YWHAQ		RPL28		PTPN11
	RPL27A			RPL14		RPL9		HNRNPA0
	AHSA1			PRPS1		HIST1H2AB		ACTN4
	GCN1			PABPN1		GCN1		SMARCA5
	PRPS1			RPL21		RCC2		PRPF6
	RPL9			NPM1		SERBP1		CHD4
	RPL24			TRIM28		PCNA		SND1
	RPL22			MATR3		RPS8		EIF3L
	SERBP1			RPS6		ZNF703		RUVBL1
	SRI			PKM				YWHAQ
				CKB				CFL1
				SNRNP70				KHDRBS1
								PPM1G
								FUS
								DDX42
								RBM4
								GLRX3
								ACLY
								ALYREF
								SMARCC1
								ANXA7
								PPP1R8
								TARDBP
								SF1
								CARM1
								RBM12
								TCP1
								PCMT1
								DDX1
								OTUB1
								ANXA6
								CRKL
								TBL3
								YWHAZ
								IMPDH
								PDCD5
								CPSF1
								PSPC1

								MDC1
								PRMT1
								API5
								XRN2
								EEF1A2
								RPRD1A
								PLRG1
								GART
								H4C1
								RPL6
								TSR1
								ACIN1
								PABPN1
								GNL3
								CNN3
								YWHAE
								DHX16
								DNAJC8
								XRCC5
								PPIA
								CSTF3
								RBM39
								PPA1
								LYAR
								SRSF6
								PRKDC
								RBM10
								SF3B3
								NSFL1C
								NT5DC1
								FXR1
								WDR46
								FAM50A
								RBBP7
								RBMX
								UBB
								CIAPIN1
								FLNA
								HSPA4L
								MCM5
								USP5
								EIF4G1
								NSUN2
								CDC73
								SERBP1
								POLR2A
								TUFM
								ANP32B
								YWHAB
								PPP1CC
								PES1
								RSL1D1
								GSR
								RPS10
								PAPOLA
								RPS3A
								SRSF2
								TOP2B

								TCOF1
								HNRNPH3
								TCERG1
								RAN
								IK
								SART3
								STIP1
								MSH6
								PPP1CA
								LDHA
								GAPDH
								HMGB1
								ATP5F1A
								DNAJA1
								CCT6A
								MAPRE1
								PFN1
								MAP4
								USP15
								SRSF10
								LUC7L3
								LDHB
								KPNA2
								ANP32E
								TRMT1
								WDR3
								SRRM1
								LSM3
								HIST1H2BK
								U2SURP
								RBM26
								XPO5
								RPS4X
								PPP1CB
								DFFA
								CPNE3
								APEX1
								CCT8
								RACK1
								TTLL12
								PWP2
								CDC37
								ZC3H14
								UPF1
								TBCB
								FTO
								PTBP2
								UBAP2L
								RAVER1
								HIST1H2BJ
								PDCD6
								UBE2L3
								DIS3
								NOSIP
								STRAP
								RBBP4
								PDLIM1
								SMNDC1

								ADSS2
								SRI
								SRSF11
								DHX38
								TRA2B
								PUF60
								PARN
								PEBP1
								RBM25
								U2AF1
								CWC27
								DCTPP1
								SAFB
								APRT
								EZR
								AHCY
								PGD
								USP39
								HNRNPLL
								HIST1H2AB
								CCT4
								ALKBH5
								DAZAP1
								DDX27
								DHX36
								CACYBP
								NOP58
								RIF1
								ZFR
								ZNF207
								SLC25A5
								RPA2
								PPP2CA
								DNTTIP2
								NIFK
								BOLA2
								SUGT1
								RPL15
								TBCA
								PRPF40A
								NUCKS1
								FOXK1
								DCUN1D5
								TAF15
								CDC40
								RPRD1B
								RNH1
								GSPT1
								MPG
								RPL4
								RPS8
								RBM3
								SF3B4
								EPRS
								NOP2
								NXF1
								DDX18
								DBN1

								SFN
								CDK4
								HMGB2
								DNAJC9
								ACTN1
								YWHAH
								PPP2R2A
								PHGDH
								ACP1
								DUT
								PRPF31
								RBM14
								C1orf52
								DDX50
								GPATCH4
								YBX1
								ZRANB2
								CSTB
								RPSA
								PABPC1
								FBL
								CRK
								CPSF6
								BAZ1B
								UTP15
								DCPS
								SRSF5
								SMAP
								LARP1
								MTREX
								BOD1L1
								MKI67
								NRDC
								YWHAG
								RPS3
								WDR36
								POLDIP3
								UCK2
								RBM22
								PSME2
								RBM8A
								SSB
								PRCC
								SON
								CTBP1
								LAS1L
								FKBP5
								FUBP3
								TPM3
								SF3A3
								PCBP3
								TOP2A
								ELOA
								CAPRIN1
								FTSJ3
								FBXO22
								PHAX
								MTHFD1

								CDV3
								FIP1L1
								NOL6
								EIF3G
								PAICS
								PREP
								RRM1
								SRSF4
								UBTF
								GDI2
								CTBP2
								ARF3
								CIRBP
								CHORDC1
								KCTD12
								QKI
								ALDOC
								NUMA1
								NUDT21
								MSH2
								SMARCE1
								C12orf10
								TEX10
								RUVBL2
								MAGOHB
								UTP3
								CCDC117
								PARK7
								CD2BP2
								PEF1
								CCDC86
								FAM98B
								NUDC
								IRS4
								SAFB2
								KRI1
								PDCD11
								GEMIN5
								TTC1
								TLE3
								GSTM3
								TIPRL
								ACTL6A
								LTA4H
								PSMF1
								HNRNPUL1
								SAE1
								RRP9
								THOC6
								GTF2E1
								EIF6
								EXOSC10
								RBM27
								DCAF13
								KPNA3
								RARS
								TP53BP1
								AHSA1

								RPL13
								RPL22
								TXN
								GRB2
								NOLC1
								RPS23
								FAM192A
								SARNP
								CBS
								HAT1
								ZC3HAV1
								PRIM2
								PRPF4
								KIF4A
								CSTF1
								RBM42
								MTA2
								RAD23B
								CHERP
								G6PD
								RECQL
								TLE4
								TIAL1
								CCT5
								RPS20
								UBE2M
								RPS12
								EBNA1BP2
								SNRPB
								NELFE
								FAM98A
								CLTA
								SRPK1
								RBM17
								RPL5
								CSE1L
								HARS
								GTPBP4
								GARS
								UGDH
								HNRNPUL2
								UBA6
								PQBP1
								RPS2
								PRDX3
								RPS16
								WDR75
								GPKOW
								CLNS1A
								SNRPA
								PGM3
								MYBBP1A
								CSTF2
								ARID1A
								DDX10
								PPP1R2
								NUP153
								SMARCA4

								NAT10
								PRPF19
								HDAC2
								HPRT1
								RPL12
								POLR2H
								KDM3B
								JPT2
								PDE12
								SNRPE
								ILKAP
								RPL10
								TPR
								THOC2
								PRPF38B
								UBXN1
								PTBP3
								LUC7L
								ENSA
								UTP14A
								TRAP1
								ANXA5
								RPS6
								RPL18
								RPL18A
								DBNL
								VRK1
								PSMD10
								EWSR1
								FXR2
								RPL11
								PHF5A
								PRPF4B
								C9orf78
								SMU1
								PRDX4
								QRICH1
								ERH
								NME1
								PCYT1A
								PDCD6IP
								SNRNP40
								FAF1
								FEN1
								MRE11
								TBCE
								AQR
								AMOT
								H3C1
								EIF3CL
								AASDHPPT
								CARHSP1
								THOC3
								COIL
								PPIE
								WRAP53
								TRA2A
								THOP1

								POLA2
								WDR43
								SKP1
								RPS11
								FDPS
								PSMD4
								CSNK2A1
								CAD
								USP14
								HEATR3
								CTNNBL1
								CBX3
								PRPF39
								CDK12
								PAPSS1
								BRD2
								CANX
								PPP4C
								CALM1
								WBP2
								DYNC1H1
								GIPC1
								MAT2A
								XPO1
								TPX2
								RGS10
								NOP16
								CFDP1
								OTUD5
								TRIP6
								ATXN2L
								CUL4B
								DKC1
								TRMT1L
								GTF3C5
								NKRF
								STAU1
								GTF2F2
								RFC1
								KRR1
								SPDL1
								SNX12
								NUDCD1
								CCT3
								RPP30
								EMD
								CORO1C
								RPL23
								CCT7
								RRS1
								VDAC2
								CAPZA1
								MIF
								CPSF7
								FHL1
								FOXC1
								TWISTNB
								PLCG1

								AIP
								RPL9
								HPF1
								ARIH1
								MEPCE
								RNPEP
								CBX5
								CAND1
								CNBP
								CCNK
								CAPZB
								VBP1
								MTPN
								DBR1
								ANXA2P2
								STEEP1
								RPL8
								PFN2
								WTAP
								FMR1
								PSIP1
								WDR18
								ZNF503
								ASNS
								HMGB3
								RPS15A
								RRP12
								ESYT1
								RANBP3
								JMJD6
								LBR
								CCDC6
								GTF2F1
								MYCBP
								POLA1
								RO60
								HDLBP
								NOL9
								SIRT1
								DEK
								CCAR2
								OLA1
								TDP2
								TRIP12
								WAPL
								PELP1
								POP1
								NOP56
								EEF1B2
								GSTO1
								SDCBP
								WDR61
								CTCF
								LANCL1
								RPS26
								YY1
								PAK2
								ATP1A1

								MAPKAPK3
								POLD2
								PWP1
								SAP18
								EIF3D
								MPHOSPH10
								PHF3
								PSMC2
								CLIC1
								RANBP1
								SNRPD2
								MTA1
								DHX8
								ABCF1
								UHRF1
								ASH2L
								ADSL
								PUS3
								RRP8
								ZC3H11A
								PPIL2
								NARS
								TARS1
								PGAM1
								RPL3
								POLR2D
								BLVRA
								PITHD1
								PHPT1
								NTMT1
								HDGFL2
								PSMG1
								CTPS1
								DACH1
								RBBP5
								ABCE1
								HMGCS1
								MMTAG2
								SAAL1
								BID
								BAZ1A
								RPS9
								PDCD4
								PIN1
								ESS2
								ZC3H4
								WDHD1
								TMA16
								EXOSC3
								ZPR1
								GTF3C2
								RPL17
								RSBN1L
								PHIP
								SUGP1
								SCAF11
								SEPHS1
								SF3B6

								CMBL
								MPST
								CNN2
								FLNB
								AFDN
								MAPRE2
								C8orf33
								PGK1
								HUWE1
								POLR2B
								NAMPT
								NUP43
								YLPM1
								LARP4B
								THUMPD1
								UBAP2
								AARS
								USP10
								NLE1
								DMAP1
								SP1
								PPIG
								SPAG7
								STAT3
								CAST
								IGBP1
								FIGNL1
								CWF19L1
								RAD23A
								COPS3
								FERMT2
								HSF1
								DYNLL1
								PPP4R3A
								CDK1
								TIA1
								MPP1
								SMC4
								RCC1
								CCAR1
								BCAS2
								SNRPB2
								LASP1
								EIF3F
								ASF1A
								NKAP
								NCOA5
								NOP14
								TSN
								TIGAR
								SRM
								MAPK1
								TCEA1
								DSP
								RSF1
								TDP1
								CMAS
								SPEN

								RNF40
								WDR6
								TCEAL4
								LARS
								NVL
								YOD1
								NELFA
								FBRS
								G3BP1
								RPL19
								CSK
								DDX23
								NANS
								MED4
								GINS1
								RTCB
								EPB41L2
								PPAT
								ADNP
								POLD1
								RPS14
								RBFOX2
								CDK6
								CDK9
								SCAF8
								EEF1D
								UCHL3
								EIF3J
								RPS27
								PGLS
								DRAP1
								LTV1
								RNF113A
								GLOD4
								NELFCD
								DNAJC7
								CDKN2A
								CUL2
								HMG20A
								DDX20
								SSR4
								RBM15
								GTF2E2
								PSMD2
								PAXBP1
								ETF1
								GATAD2B
								RAB5C
								AIMP2
								BCCIP
								INTS3
								DUS3L
								PLAA
								RRP1B
								RTF2
								SLTM
								STAG2
								YJU2

								TFIP11
								USP48
								DHX57
								CDK5
								MAPKAPK2
								ISY1
								EIF1AY
								CDC34
								KDM1A
								SCML2
								UAP1
								EIF1
								RAB1A
								VCL
								S100A11
								PSME1
								PRPS1
								NUDCD2
								CPSF2
								EIF2S1
								RPN1
								GTF3C1
								OGFOD1
								IRF2BP2
								RPL23A
								EIF3E
								RTF1
								RAB7A
								FRG1
								EIF4EBP1
								FSCN1
								XRCC1
								DNAJA2
								RRP15
								DARS
								NCOR1
								RBM34
								EYA3
								WARS
								TWF1
								UBE2G1
								GLO1
								PARG
								SNCA
								ST13
								EXOSC7
								UBA3
								RTRAF
								RBBP6
								UBE2S
								GINS3
								GSK3B
								PSMC1
								SPCS2
								GPS1

Each column shows a list of proteins ranked according to the total spectral count from three replicates. High to low scores are ranked top to bottom respectively. Proteins detected in only one replicate, common contaminants (keratin, tubulin, etc.) and naturally biotinylated proteins (carboxylases) are excluded.

Supplementary Table 2. Proteins appearing in lists from all helicase/SRSF1 pairs.

Ribosomal	Splicing	Other
EEF1A1	DDX3X	AHSA1
EEF2	DDX17	ATP5F1A
RACK1	DDX39B	BCLAF1
RPL10	DDX5	CAD
RPL11	DHX15	CFL1
RPL13	HNRNPA0	DHX9
RPL17	HNRNPA1	FASN
RPL18A	HNRNPA2B1	GAPDH
RPL19	HNRNPA3	H3C1
RPL22	HNRNPAB	H4C1
RPL23	HNRNPD	HIST1H2AB
RPL27A	HNRNPDL	HIST1H2BK
RPL4	HNRNPF	HSP90AB1
RPL7	HNRNPH1	HSPA1A
RPS11	HNRNPH2	HSPA8
RPS14	HNRNPK	HSPD1
RPS15A	HNRNPL	MCM7
RPS16	HNRNPR	MTHFD1
RPS2	HNRNPU	PARP1
RPS20	KHSRP	PCBP2
RPS23	PCBP1	PCMT1
RPS3	RBMX	PHGDH
RPS3A	SFPQ	PRDX1
RPS4X	SRSF1	PRDX6
RPS9	SRSF2	PRKDC
	SRSF3	RAN
	SRSF7	SLC25A5
	SYNCRIP	SLC25A6
		TCP1
		UBB

The columns show the 84 proteins common to all the split-APEX pairs of SRSF1 and helicases, classified according to whether they were involved in ribosome-associated processes (translation and biogenesis), splicing or other processes.

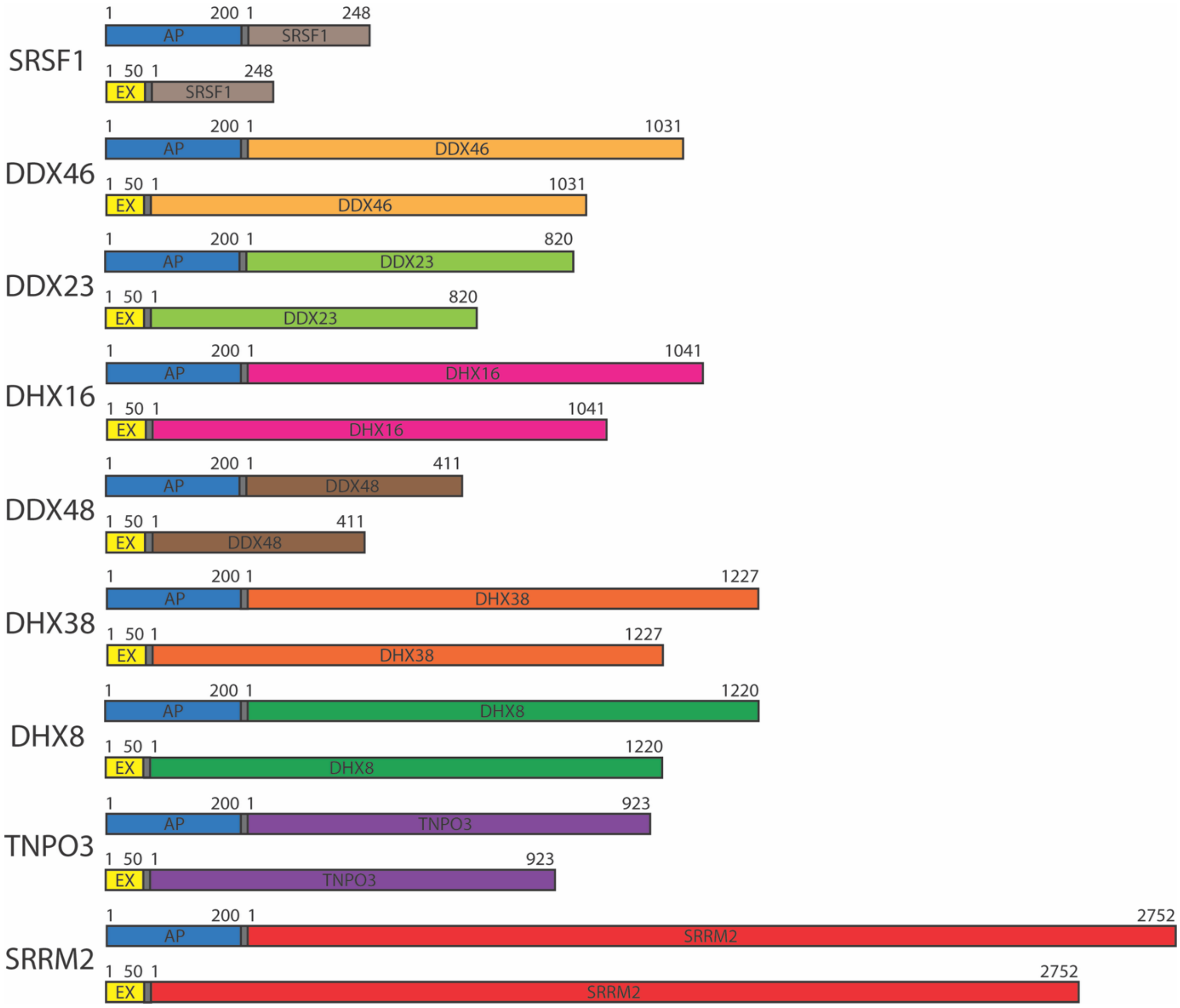

Supplementary Figure 1.

Diagrams of the split-APEX2 fusion constructs. Proteins are shown fused to either the AP or EX fragments via a linker of nine amino acids (grey).

A

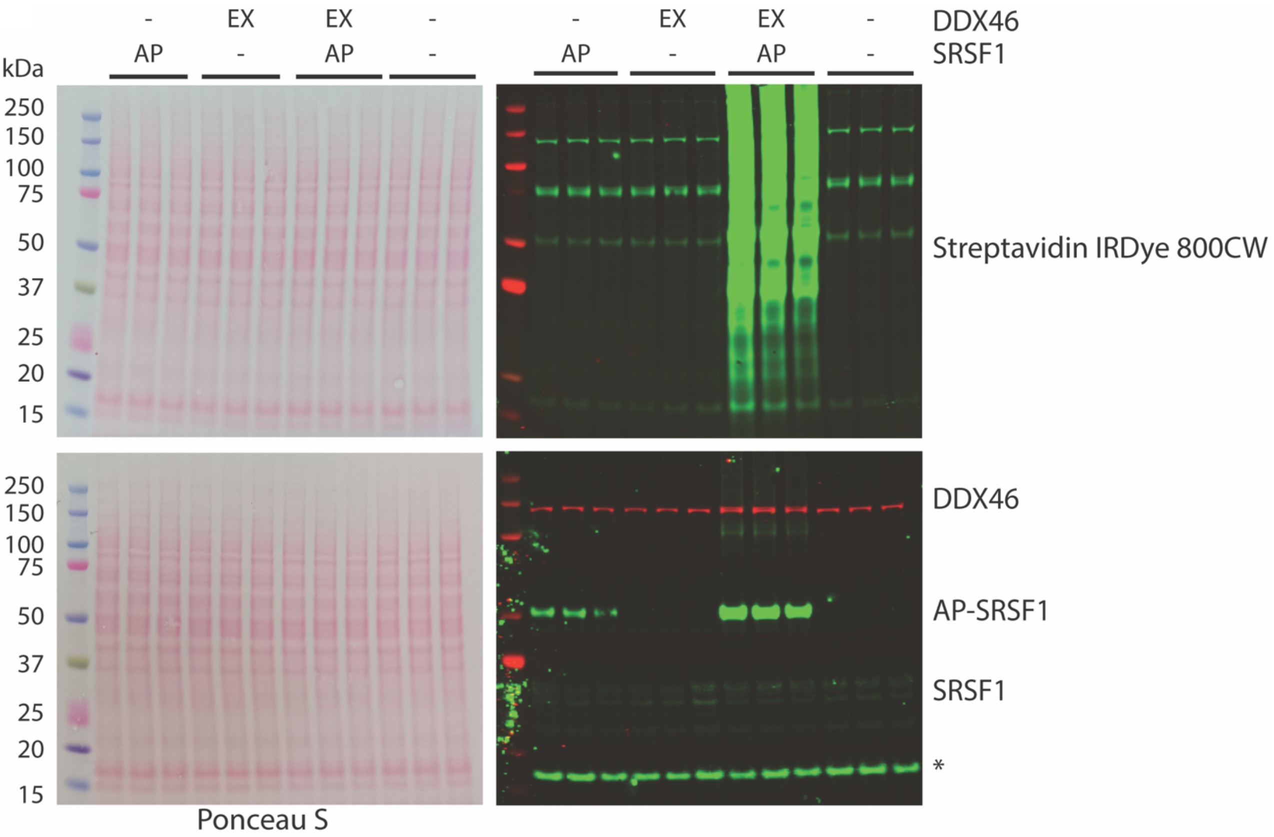

- EX EX -
AP - AP -
- EX EX -
AP - AP -
DDX46
SRSF1
kDa
250
150
100
75
50
37
25
20
15
Streptavidin IRDye 800CW
DDX46
AP-SRSF1
SRSF1
*
Ponceau S

B

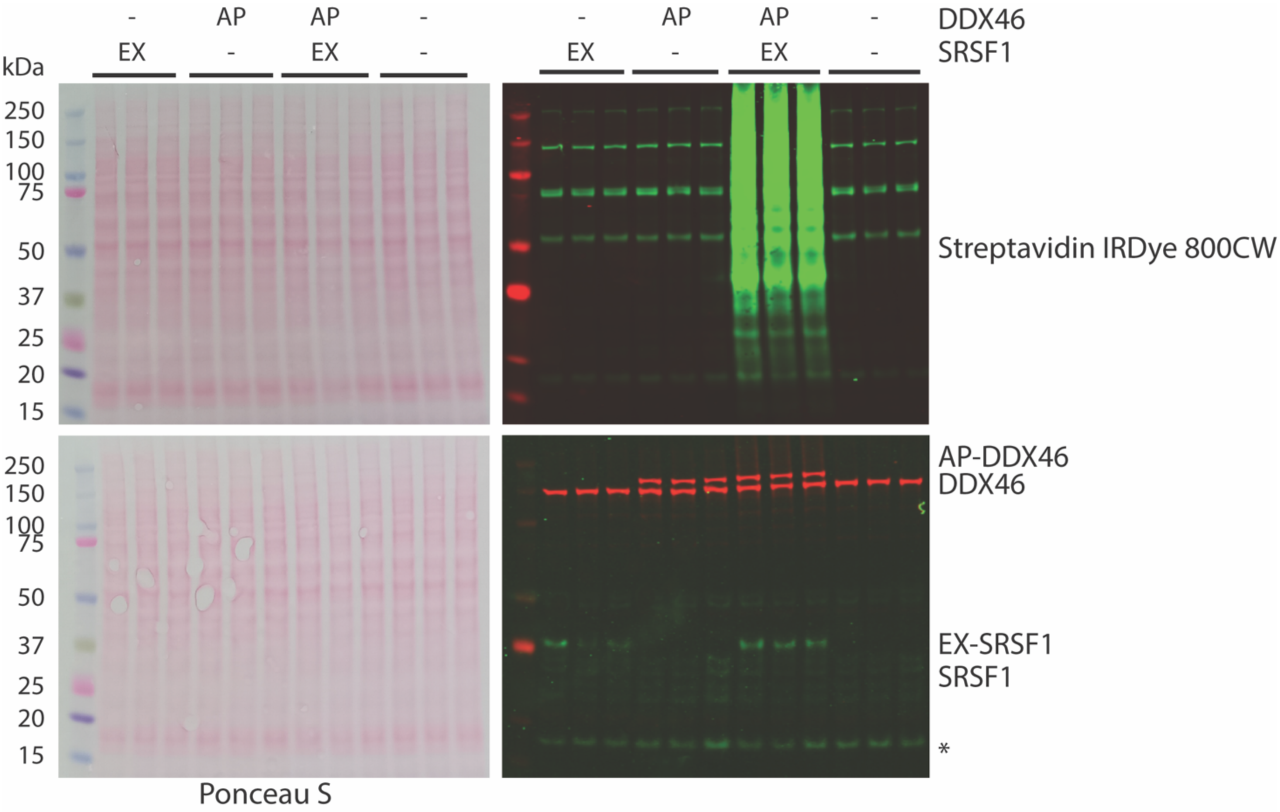

- AP AP -
EX - EX -
- AP AP -
EX - EX -
DDX46
SRSF1
kDa
250
150
100
75
50
37
25
20
15
Streptavidin IRDye 800CW
AP-DDX46
DDX46
EX-SRSF1
SRSF1
*
Ponceau S

Supplementary Figure 2.

Biotinylation profile of single transfections. (A) HEK293T cells were transfected with the plasmids encoding EX-DD46, AP-SRSF1 or both. Proximity biotinylation was conducted 24 h after transfection. Following lysis, 40 µg of total protein from each sample were analysed by SDS-PAGE and western blotting. All proteins were detected by Ponceau S staining of the transfer membranes (left-hand panels). Biotinylation was detected using Streptavidin IRDye 800CW (upper-right panel). The expression of EX-DDX46 and AP-SRSF1 was monitored using anti-DDX46 (red) and anti-SRSF1 (green) antibodies (lower-right panels). The asterisk indicates non-specific binding of goat anti-mouse secondary antibody. (B) As A, but with expression of AP-DDX46 and EX-SRSF1.

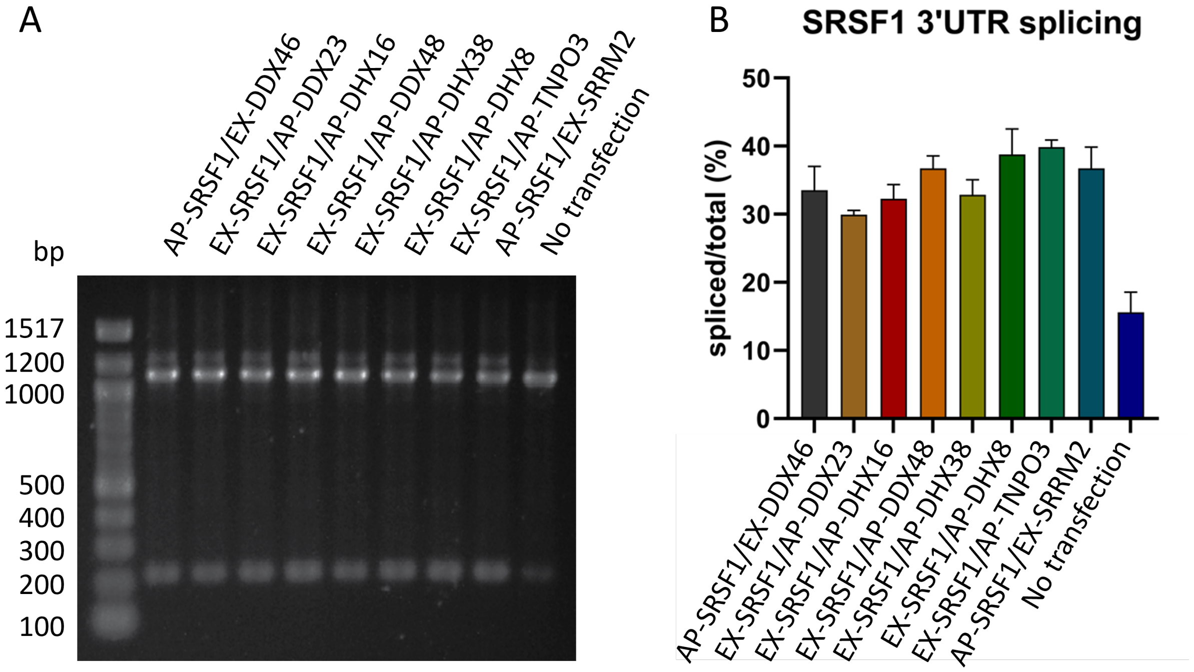

Supplementary Figure 3.

Activity of SRSF1 fusions in auto-regulation of splicing. (A) HEK293T cells were transfected with plasmids encoding the split APEX2 protein pairs, the total RNA was extracted, cDNA was generated and the presence of a cryptic intron at SRSF1 3'UTR RNA was detected by PCR. Splicing of this intron is enhanced by SRSF1, and leads to nonsense-mediated decay. The upper band at 1,136 base pairs derives from unspliced RNA, and the lower band at 216 base-pairs is derived from spliced RNA. (B)The bands were quantified and the ratio of the intron excision versus the total PCR product was plotted. All transfections were performed in triplicates. Error bars show standard deviation.

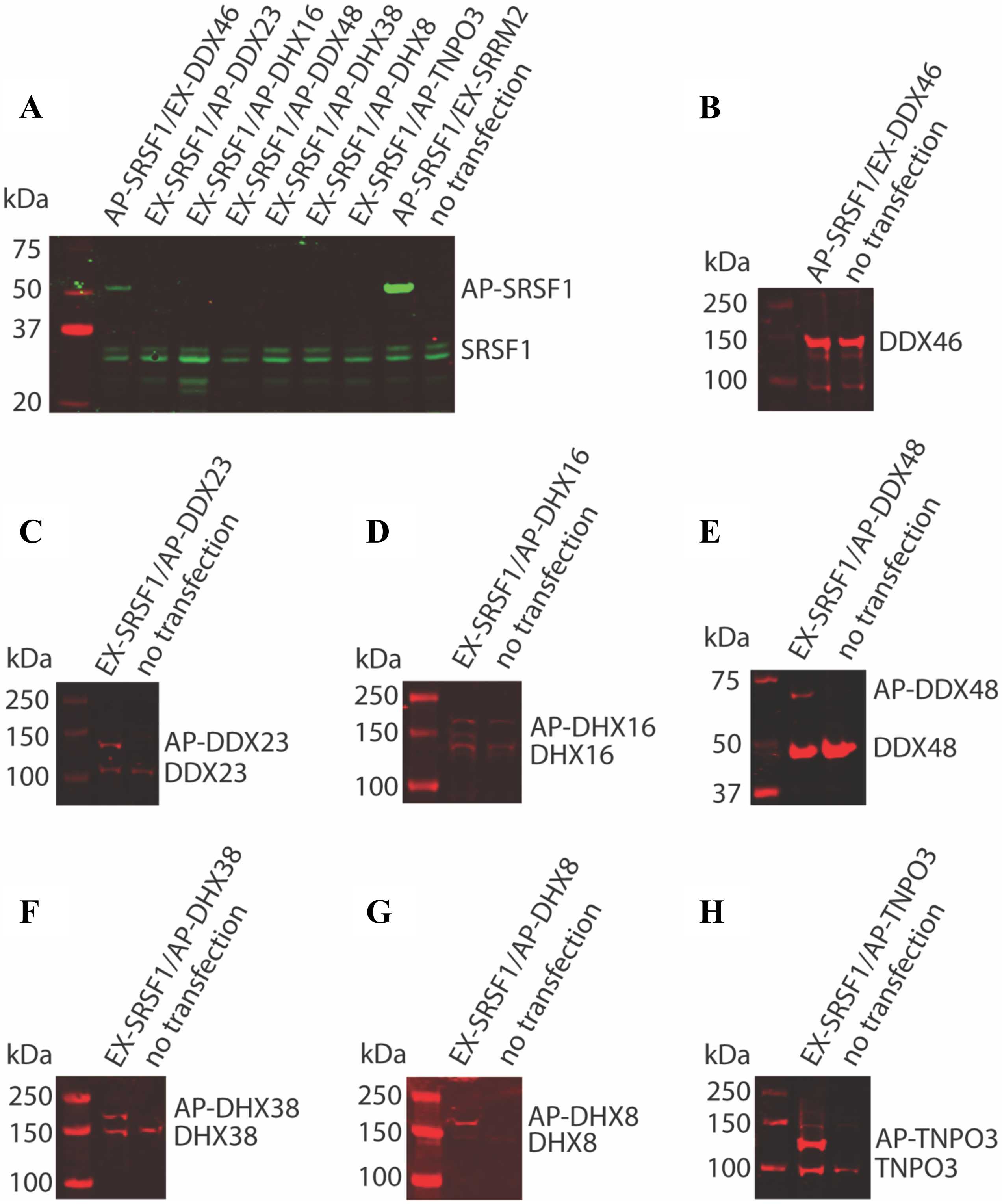

Supplementary Figure 4.

Expression of the fused proteins. (A) Expression of SRSF1 fusions when the combinations of plasmid that produced successful biotinylation (Figure 1) were co-transfected into HEK293T cells. In each case, 40 µg of total protein was analysed by SDS-PAGE and western blotting. (B) - (H), analysis of the expression of the fused helicase or TNPO3.

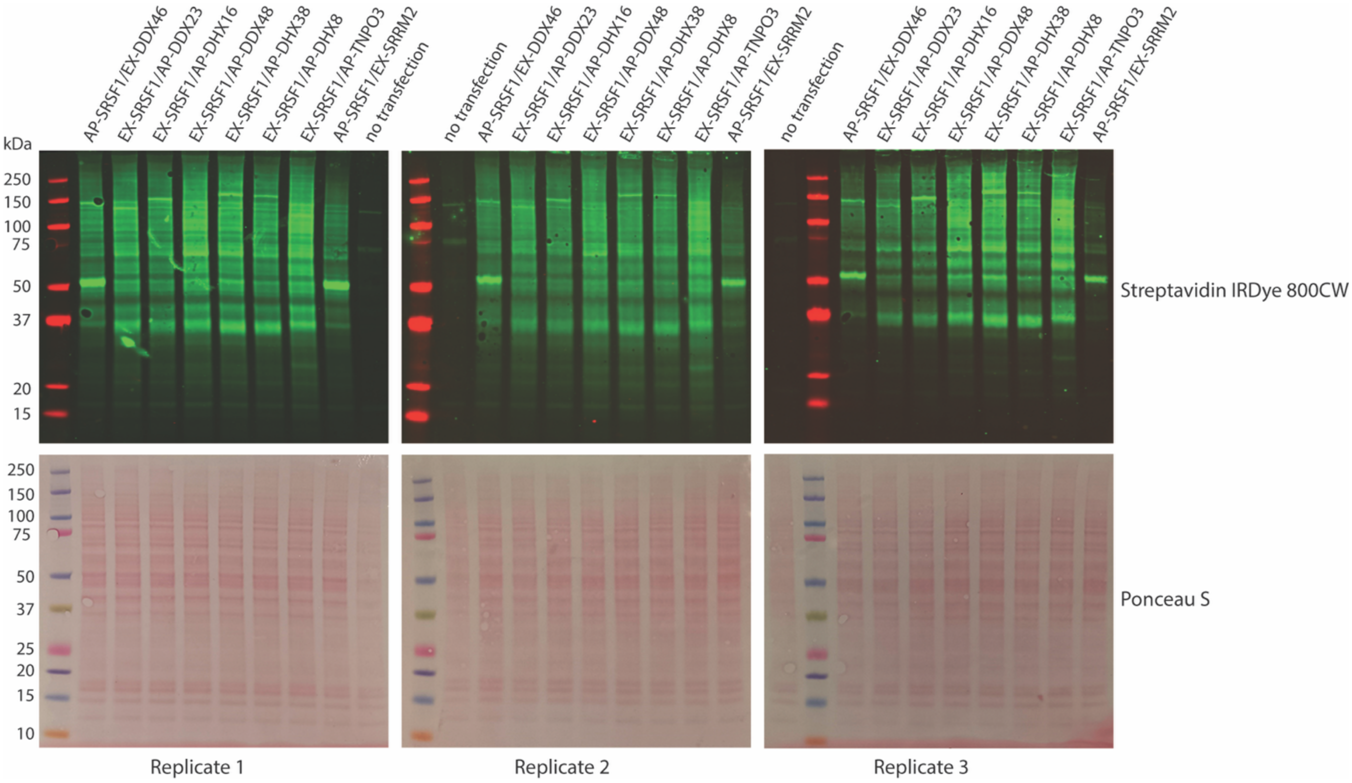

Supplementary Figure 5.

Characterisation of the replicate samples sent for mass spectrometry. HEK293T cells were transfected with the selected combinations of plasmids expressing the split-APEX fusion proteins, and proximity biotinylation was conducted 24 h after transfection. Following lysis, 40 µg of total protein from each sample were analysed by SDS-PAGE and western blotting. Biotinylation was detected using Streptavidin IRDye 800CW (top panels) and all proteins were detected by Ponceau staining of the transfer membrane (lower panels).

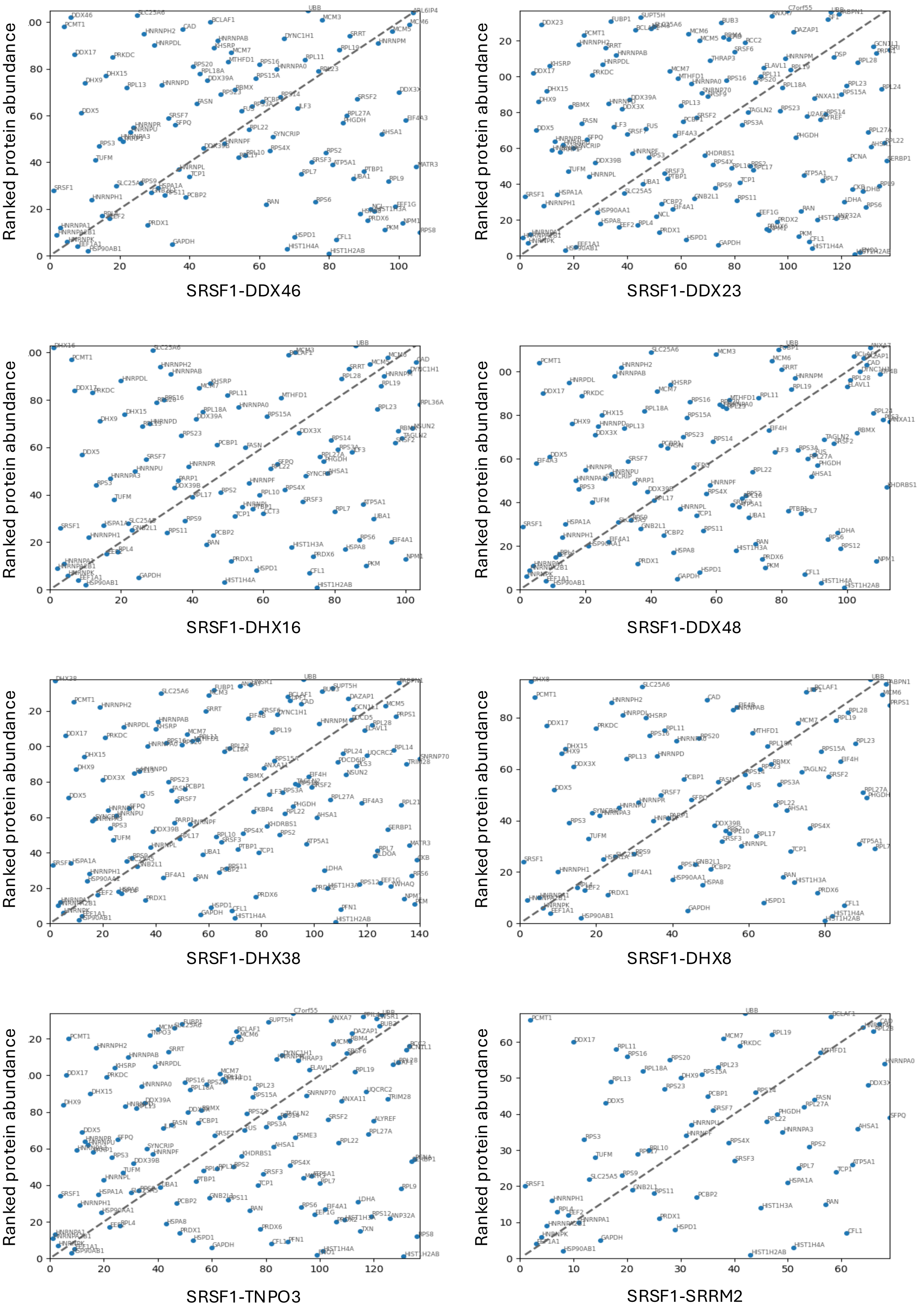
Ranked protein abundance
SRSF1-DDX46
SRSF1-DDX23
SRSF1-DHX16
SRSF1-DDX48
SRSF1-DHX38
SRSF1-DHX8
SRSF1-TNPO3
SRSF1-SRRM2

Supplementary Figure 6.

Comnparisons of the rank of each of the proteins detected in the split-APEX2 datasets with the abundance of the protein in HeLa cells. The abscissa shows the rank of each protein, as labeled, detected in the split-APEX experiment and the ordinate shows the ranked concentrations of proteins in HeLa cells (Hein *et al.*, 2015).
